# Extracellular vesicles released by host epithelial cells during *Pseudomonas aeruginosa* infection function as homing beacons for neutrophils

**DOI:** 10.1186/s12964-024-01609-7

**Published:** 2024-06-21

**Authors:** Rajalakshmy Ayilam Ramachandran, Andrew Lemoff, Danielle M. Robertson

**Affiliations:** 1 The Departments of Ophthalmology, UT Southwestern Medical Center, Dallas, TX USA; 2 The Departments of Biochemistry, UT Southwestern Medical Center, Dallas, TX USA; 3 The Department of Ophthalmology, UT Southwestern Medical Center, 5323 Harry Hines Blvd, 75390-9057 Dallas, TX USA

**Keywords:** *Pseudomonas aeruginosa*, Proteomics, Extracellular vesicles, Cornea, Lung, Epithelium, Neutrophils

## Abstract

**Background:**

*Pseudomonas aeruginosa* (PA) is an opportunistic pathogen that can cause sight threatening infections in the eye and fatal infections in the cystic fibrosis airway. Extracellular vesicles (EVs) are released by host cells during infection and by the bacteria themselves; however, there are no studies on the composition and functional role of host-derived EVs during PA infection of the eye or lung. Here we investigated the composition and capacity of EVs released by PA infected epithelial cells to modulate innate immune responses in host cells.

**Methods:**

Human telomerase immortalized corneal epithelial cells (hTCEpi) cells and human telomerase immortalized bronchial epithelial cells (HBECs) were treated with a standard invasive test strain of *Pseudomonas aeruginosa*, PAO1, for 6 h. Host derived EVs were isolated by qEV size exclusion chromatography. EV proteomic profiles during infection were compared using mass spectrometry and functional studies were carried out using hTCEpi cells, HBECs, differentiated neutrophil-like HL-60 cells, and primary human neutrophils isolated from peripheral blood.

**Results:**

EVs released from PA infected corneal epithelial cells increased pro-inflammatory cytokine production in naïve corneal epithelial cells and induced neutrophil chemotaxis independent of cytokine production. The EVs released from PA infected bronchial epithelial cells were also chemotactic although they failed to induce cytokine secretion from naïve HBECs. At the proteomic level, EVs derived from PA infected corneal epithelial cells exhibited lower complexity compared to bronchial epithelial cells, with the latter having reduced protein expression compared to the non-infected control.

**Conclusions:**

This is the first study to comprehensively profile EVs released by corneal and bronchial epithelial cells during *Pseudomonas* infection. Together, these findings show that EVs released by PA infected corneal and bronchial epithelial cells function as potent mediators of neutrophil migration, contributing to the exuberant neutrophil response that occurs during infection in these tissues.

**Supplementary Information:**

The online version contains supplementary material available at 10.1186/s12964-024-01609-7.

## Background

*Pseudomonas aeruginosa* (PA) is a gram negative, non-spore forming opportunistic pathogen that can cause infection in immunocompromised individuals [1]. In the eye, PA can invade the otherwise healthy cornea when there is a breach in innate defense barriers through injury or during contact lens (CTL) wear [2, 3]. Indeed, studies show that CTL wear is the leading cause of infectious keratitis and when severe, can lead to permanent vision loss [4]. Multiple pathogenic mechanisms are involved in aiding pathogen entry into the otherwise resistant cornea [2, 4]. Early work using the rabbit CTL model showed that wear of low oxygen transmission CTLs promoted lipid raft formation and internalization of PA into the corneal epithelium [2, 5, 6]. Once internalized, PA is then able to form membrane blebs in corneal epithelial cells, providing a safe haven for replication and survival [7, 8]. 

CTLs serve as vectors for transmission of PA to the eye. Our laboratory has shown that when trapped between the CTL and the cornea, PA exploits the robust neutrophil response to enhance colonization of the CTL, leading to severe infection and corneal liquefaction [9–11]. More recently, we have shown that extracellular vesicles (EVs) released by PA are enriched in virulence factors that stimulate cytokine production by corneal epithelial cells [12]. This includes IL-8, a major chemotactic stimulus for neutrophils. While PA-derived EVs stimulated neutrophil migration, the soluble free proteins released by PA attenuated neutrophil killing. Together, these data show that factors released by the bacteria themselves are able to effectively disarm innate host immune responses.

In addition to the eye, PA is a major respiratory pathogen and is associated with significant morbidity and mortality in the cystic fibrosis airway [13, 14]. Unlike acute corneal disease, the PA-infected airway can exhibit both an acute and chronic disease phenotype [15]. This is due in part to biofilm formation that is accelerated by the exuberant neutrophil response. Other factors contributing to the susceptibility of the lung to PA invasion include impaired mucociliary clearance, defects in antimicrobial peptides and other antimicrobial molecules such as nitric oxide and glutathione, the increased availability of bacterial attachment receptors on the lung surface, and reduced ingestion of bacteria by immune cells [13, 16]. 

Extracellular vesicles (EVs) are nanoscale membrane embedded particles that are released by most cells into the extracellular milieu [17, 18]. It is well established that the biomolecular cargo transported by EVs have a profound role in regulating disease pathogenesis in various microbial infections [19]. Indeed, both pathogen and host-derived EVs carry virulence factors and bacterial components that mediate disease outcome by regulating host immunity [19]. Prior work investigating the role of EVs during PA infection have been focused on pathogen-derived vesicles. They have been shown to regulate antibiotic resistance, bacterial quorum sensing properties, and host adaptive immunity via pathogen associated molecular factors such as LPS, flagellin, and CpG DNA [20–24]. In the cornea, human tear lysozyme was shown to induce PAO1 EV production, and these EVs elicited immune responses in the mouse cornea [25]. Similarly, clinical isolates of PA from CF patients were also able to regulate immune responses and circumvent host immune evasion strategies [26–28]. 

Currently, there are no studies on the composition and functional role of host-derived EVs during PA infection of the cornea or lung. To address this major gap, we have compared the proteomic profiles of EVs derived from human corneal and bronchial epithelial cells during PA infection. We further investigated the effects of these EVs on the host cell immune response and PA intracellular survival. Since neutrophils play a key role in the etiology of infection for both tissues, understanding how infection alters the composition and function of EVs released from each cell type is essential for elucidating the complex pathophysiology of corneal and pulmonary disease.

## Materials and methods

### Cell culture

The human telomerase immortalized corneal epithelial (hTCEpi) cell line used in this study was previously developed and characterized by our laboratory [29]. Human bronchial epithelial cells (HBECs) were a generous gift from Dr. Jerry Shay (Department of Cell biology, UT Southwestern Medical Center, Dallas, Texas) [30]. Both cell lines were routinely maintained in serum-free keratinocyte basal media (KBM) containing supplements (KGM, VWR, Radnor, PA) and 10% Pen/Strep/Amphotericin B (Lonza, Walkersville, MD). KGM was supplemented with additional CaCl_2_ (Calcium Chloride Solution, 0.5 M, VWR, Radnor, PA) to a final concentration of 0.15 mM. HL-60 cells were purchased from ATCC (Manassas, VA) and maintained in Isocove’s Modified Dulbecco’s Medium (IMDM, ATCC) with 20% fetal bovine serum (FBS, Sigma-Aldrich, St. Louis, MO) and 10% Pen/Strep/Amphotericin B. Growth medium containing 1.3% dimethyl sulfoxide (DMSO) was used for 5 days to differentiate HL-60 cells into neutrophil-like cells. All cells were maintained at 37 °C and 5% CO_2_. For studies involving primary neutrophils, blood was collected from healthy volunteers via venipuncture. All procedures were approved by the Institutional Review Board at UT Southwestern Medical Center. Prior to collection, written informed consent was obtained from each subject. Primary neutrophils were isolated using magnetic isolation per the manufacturer’s recommended instructions (Miltenyi biotech, Bergisch Gladbach, Germany). Residual erythrocytes were depleted using MACSxpress® Erythrocyte Depletion Kit (Miltenyi, Bergisch Gladbach, Germany).

### EV isolation

**Fig. 1:**
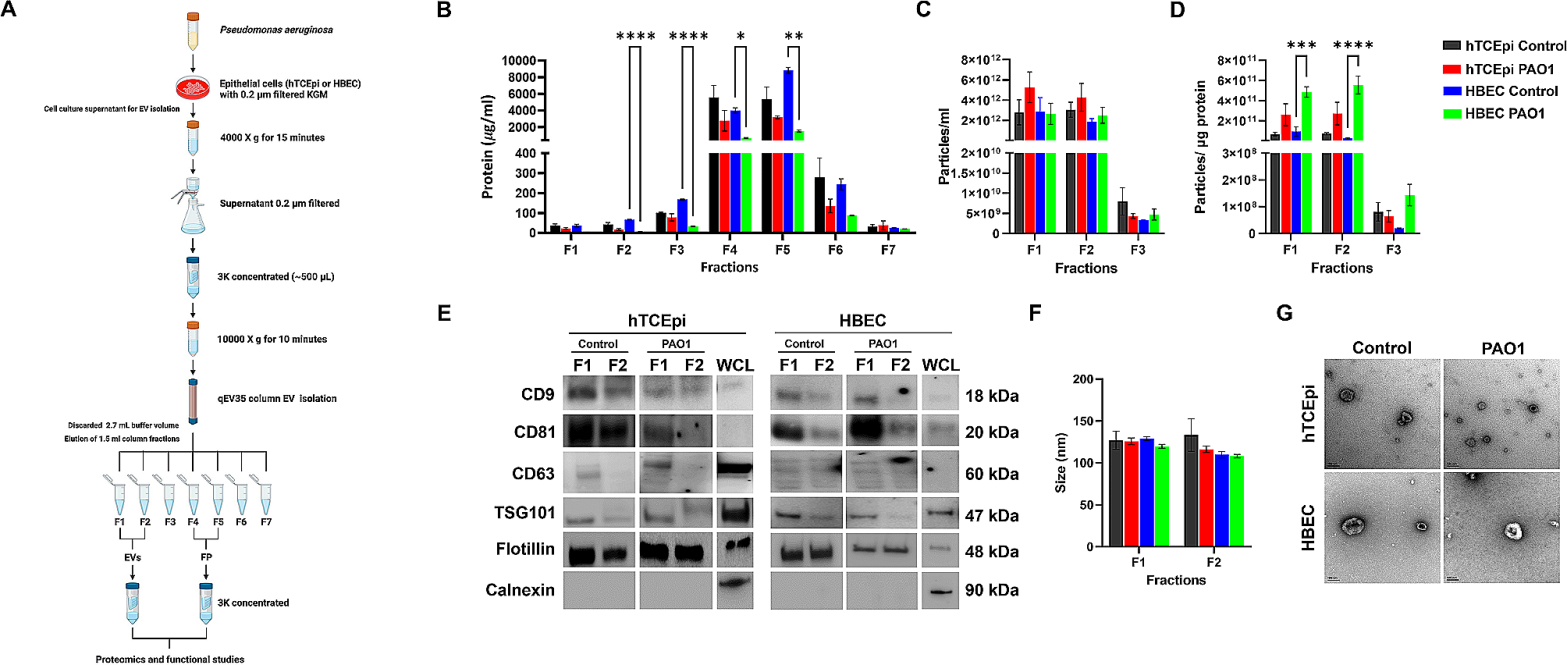
F1 and F2 contained pure EVs. EVs were isolated from uninfected (control) and PA infected (PAO1) human telomerase immortalized corneal epithelial (hTCEpi) cells and human telomerase immortalized bronchial epithelial cells (HBECs).(**A**) EV isolation protocol. (**B**) The protein concentration of each fraction was measured using a BCA assay. Protein concentration was less in early fractions and highest in F4 and F5. (**C**) Particle concentration for F1– F3 measured using a nanoparticle tracking analyzer (NTA). (**D**) EV fraction purity for F1– F3 measured by calculating particle / µg protein ratio (PP ratio). F1 and F2 showed highest particle concentration with superior PP ratio. (**E**) Western blot for known EV markers CD9, CD81, CD63, TSG101, and flotillin. Calnexin was used as a negative EV marker. EV positive markers, but not calnexin, were detected in F1 and F2. Whole cell lysates (WCL) from healthy control hTCEpi cells and HBECs were also included. (**F**) F1- F2 particle size measured by NTA. (**G**) Transmission electron microscopy (TEM) images of pooled F1 and F2 collected from non-infected controls and PA infected epithelial cells. NTA and TEM confirmed the presence of EVs < 200 nm in F1 and F2. TEM image scale bar 100 nm. Data presented as mean ± standard deviation. *N* = 3 independent samples, *p < 0.05, **p < 0.01, ***p < 0.001, ****p < 0.0001. One-way ANOVA with Tukey post host multiple comparison test

A standard invasive strain of *Pseudomonas aeruginosa*, PAO1, was used for infecting hTCEpi cells and HBECs at a concentration of ∼ 1 multiplicity of infection (MOI) for 6 h in 50 mL of serum and antibiotic free KGM. Cell culture supernatants were collected from healthy control and PAO1 infected cells and EVs were isolated as detailed in Fig. 1A. In brief, the cell culture supernatants were centrifuged at 4000Xg for 15 min and the bacterial pellet was discarded. Supernatants were then subjected to filtration using a 0.2 μm filter and concentrated using 3 K concentrators (Pierce protein concentrator PES 3 K, Thermo Fisher Scientific, Rockford, IL) to remove bacteria. Concentrated supernatant was further subjected to centrifugation at 10000Xg for 10 min to remove larger vesicles. Approximately 500 µL of the concentrated culture supernatants were passed through a size exclusion column (qEV35 columns, Izon Biosciences, Cambridge, MA) and fractions were eluted using phosphate buffered saline (PBS). After the void volume of 2.7 µl for the qEV35 column was eluted, a total of seven 1.5 µl fractions were collected. The presence of pure and enriched EVs in fractions F1 and F2 was confirmed by measuring the particle/protein ratio (PP ratio) and western blot analysis for known markers. F1 and F2 were then pooled, concentrated and used as extracellular vesicles (EVs). Since fractions F4 and F5 showed the highest protein concentration with very low particle numbers, these were similarly pooled, concentrated and analyzed as free proteins (FPs). After isolation, EVs and FPs were stored at -80 °C until further analysis or use in functional studies. This protocol was based on an established size exclusion chromatography protocol that has been used for isolating host derived EVs from infection models [31]. As the host derived and bacterial EVs share similar size, it is possible that there could be traces of bacteria derived EVs also present in the sample preparation. However, in our prior study on bacteria derived EVs, the total protein concentration and number of EV particles in F1 and F2 were significantly lower, despite a 5 fold greater volume of sample, compared to the host derived EVs reported here [12]. Hence, the EVs isolated in the current study are most likely enriched with host derived EVs. Published protocols on bacterial infection and host derived EV studies use 0.2 μm filtration to remove bacteria [32]. Additionally, the concentrated supernatants were tested by plating to confirm the absence of live bacteria.

### EV characterization

EV protein concentration was measured using either a BCA assay (Thermo Fisher Scientific, Rockford, IL) or a Qubit protein assay (Thermo Fisher Scientific). A nanoparticle tracking analyzer (NTA) (NanoSight NS300, Malvern Panalytical, Worcestershire, UK) configured with a 532 nm laser and a high sensitivity scientific CMOS camera was used to assess particle concentration and size. EV fractions were diluted in PBS according to the manufacturer’s recommendation. The samples were analyzed for 30 s, 5 separate times, with a camera level of 15. The data were analyzed using TA 3.4 Build 3.4.4 software. Data from a minimum of 3 independent measurements for each run were used for quantification. EVs were lysed using RIPA and electrophoresed through a 4–12% gradient gel (Bio-Rad, Hercules, CA). The proteins were then blotted onto polyvinylidene difluoride (PVDF) membranes (Bio-Rad, Hercules, CA) and subject to western blotting (WB). All primary antibodies were purchased from Abcam (Cambridge, MA), and secondary antibodies were from Bio-Rad (Hercules, CA). The following antibodies were used: CD9 #ab236630, CD81 #ab109201, CD63 #ab134045, TSG101 #ab83, flotillin #ab133497 and calnexin #ab112995. The secondary antibodies included an anti-rabbit HRP #1,706,515 and anti-mouse HRP #1,706,516. As a control, whole cell lysates (WCL) of healthy hTCEpi cells or HBECs were used for these markers in the western blot. For morphological analysis of EVs, transmission electron microscopy (TEM) was used. EV samples were mixed 1:1 with 2.5% glutaraldehyde in PBS for 30 min. A 400 mesh copper grid with carbon-coated formvar film was incubated with 10 µl of the sample for 20 min. After washing with MiliQ water, the grid was stained using 2% uranyl acetate for 10 s, followed by 3 washes with MilliQ water. An FEI Tecnai G2 Spirit Biotwin transmission electron microscope was used to capture EV images at the Electron Microscopy Core Facility at UT Southwestern Medical Center.

### Mass spectrometry and proteomic analysis

Mass spectrometry was performed at the Proteomics Core Facility at UT Southwestern Medical Center. Briefly, equal total protein amounts for all samples were mixed with Laemmli buffer (Bio-Rad, Hercules, CA) and subjected to SDS-PAGE. Samples were run approximately 1 cm into the separating gel and were then digested overnight with trypsin (Pierce Biotechnology, Rockford, IL) at 37^o^C followed by reduction with 20 mM dithiothreitol (DTT) at 37^o^C for 1 h and alkylation with 27.5 M iodoacetamide in the dark for 20 min. Solid-phase extraction cleanup was performed using Oasis HLB plate (Waters, Milford, MA) and the resulting samples were injected into an Orbitrap Fusion Lumos (Thermo, Waltham, MA) mass spectrometer (MS) coupled to an Ultimate 3000 RSLC-Nano liquid chromatography system (Thermo). Samples were injected onto a 75 μm i.d., 75-cm long EasySpray column (Thermo) and eluted with a gradient from 0 to 28% buffer B over 90 min. Buffer A was 2% (v/v) ACN and 0.1% formic acid in water, and buffer B was 80% (v/v) ACN, 10% (v/v) trifluoroethanol, and 0.1% formic acid in water. The mass spectrometer operated in positive ion mode with a source voltage of 1.5 kV and an ion transfer tube temperature of 275 °C. MS scans were acquired at 120,000 resolution in the Orbitrap and up to 10 MS/MS spectra were obtained in the ion trap for each full spectrum acquired using higher-energy collisional dissociation (HCD) for ions with charges 2–7. After an ion was selected for fragmentation, dynamic exclusion was set for 25 s. Proteome Discoverer v2.4 SP1 (Thermo) was used for analyzing raw MS data, with peptide identification performed using Sequest HT. Human and *Pseudomonas aeruginosa* protein databases were searched in UniProt. Fragment and precursor tolerances of 10 ppm and 0.6 Da were specified, and three missed cleavages were allowed. Carbamidomethylation of Cys was set as a fixed modification, with oxidation of Met set as a variable modification. A 1% false-discovery rate (FDR) cutoff was set for all peptides. FunRich Bioinformatics tool was used to represent data from the proteomics experiments. PANTHER.db was used for gene ontology (GO) analysis.

### Lactate dehydrogenase (LDH) assay

A colorimetric based LDH assay (Abcam, Waltham, MA) was performed to detect cytotoxicity per the manufacturer’s instruction. In brief, hTCEpi cells and HBECs were seeded onto a 96 well plate overnight in serum and antibiotic free KGM. Cells were treated with 25 µg/mL of EVs or FPs for 24 h. The cell culture supernatant was mixed with the LDH reaction mix and absorbance was measured at 450 nm. Data was expressed as percent viability, determined by subtracting cytotoxicity from 100%. Data is shown from 3 independent samples each run with 3 technical replicates.

### Gentamicin invasion assay

A gentamicin invasion assay was used to measure intracellular levels of bacteria. hTCEpi cells and HBECs were seeded and allowed to adhere overnight in serum and antibiotic free KGM. Cells were then treated for 24 h with 25 µg/ml of EVs or FPs. At the end of the treatment period, the cells were infected with the overnight culture of PAO1 at a cell: bacteria ratio of 1:500 and incubated for 2 h. The inoculum was measured by optical density using a spectrophotometer and verified using standard plate counts. At the end of the incubation period, the cells were treated with 200 µg/ml gentamicin in KGM for 1 h, followed by lysis with 0.25% Triton X 100 and plating to enumerate bacterial count. The experiments were repeated at least 3 independent times with technical triplicates for each group.

### Cytokine ELISA

Pure EVs or FPs from each group were included in the analysis to quantify the cytokines present in the absence of cells and are represented as NT-EV and NT-FP for each group. hTCEpi cells and HBECs were treated with 25 µg/ml EVs or FPs for 24 h. At the end of the treatment period, cell culture media was collected and stored at -80^°^C until analysis. IL-6 and IL-8 cytokine levels were measured using ELISAs (R & D system, Minneapolis, MN). ELISAs were performed per the manufacturer’s instruction. Samples were thawed on ice and appropriate dilutions for each assay were determined in separate experiments. In brief, the standards and samples were added into the cytokine antibody pre-coated wells. At the end of the incubation period, any unbound samples were washed away and an enzyme-linked polyclonal antibody to detect the cytokine of interest was added. After washing, the wells were incubated with a substrate against the enzyme. A BioTek plate reader (Winooski, VT) at 450 nm was used to measure color development. Cytokine concentrations were determined using a standard curve. Data is presented from 3 independent biological samples per group.

### Chemotaxis

Human peripheral blood neutrophils and DMSO differentiated HL-60 cells were used for this assay. Neutrophil migration was assessed using a commercially available Boyden chamber fluorometric assay (Cell Biolabs, San Diego, CA). In brief, the DMSO differentiated HL-60 cells or human primary neutrophils were seeded onto the top chamber. Twenty-five µg/ml EVs or FPs were added in the bottom chamber. After a two hour incubation, the migrated cells in the bottom chamber were lysed and fluorescence was measured using a BioTek plate reader (Winooski, VT). Data is presented from 3 independent biological samples per group.

### Immunofluorescence and flow cytometry

Differentiated HL-60 cell viability was measured using trypan blue (Sigma-Aldrich, St. Louis, MO). For each sample, 1 × 10^6^ viable cells were used. After treatment with 25 µg/ml EVs or FPs for one hour, cells were stained with fluorescent dye tagged antibodies from BD Biosciences (Franklin Lakes, NJ). Antibody concentrations were used as recommended on the manufacturer’s data sheet. The flow cytometry staining panel included the viability dye 7’AAD (5 µl/test) and the following primary antibodies: mouse monoclonal anti-CD35 BV421 clone E11 (5 µl/test), mouse monoclonal anti-CD66b FITC clone G10F5 (20 µl/test), and mouse monoclonal anti-CD11b PE clone ICRF44 (20 µl/test). Appropriate isotype controls included a FITC Mouse IgM clone G155-228 (20 µl/test) and PE Mouse IgG1 clone MOPC-21 (20 µl/test). Cells were kept at 4^°^C throughout the staining protocol. The cells were incubated for 20 min with respective antibody cocktails prepared in staining buffer (BD Biosciences), washed 3 times in PBS and analyzed using a Sony SH800 flow cytometer (Sony Biotechnology, San Jose, CA). A minimum of 10,000 total events were counted for each sample and analyzed using FlowJo version 10 software. Data is presented from 3 independent biological samples per group. The gating strategy for HL-60 cells were forward and side scatter followed by doublet discrimination and live cells (Supplementary Fig. 1G).

### Bacterial killing by neutrophils

DMSO-differentiated HL-60 cells were treated with 25 µg/ml EVs or FPs for 1 h in antibiotic free medium. At the end of the incubation period, cells were incubated with PAO1 at a neutrophil: bacteria ratio of 2:1 in a static condition for 1 h at 37^°^C. The bacterial killing capacity was quantified by enumerating the CFU in each group by standard plating onto tryptic soy agar (TSA, Sigma-Aldrich, St. Louis, MO). Data is presented from 3 to 4 independent samples. Technical duplicates or triplicates were included in the plate count for each group.

### Neutrophil respiratory burst

Neutrophil respiratory burst was measured by flow cytometry using a commercially available kit (Abcam, Waltham, MA). DMSO-differentiated HL-60 cells were stained with dihydrorhodamine 123 and incubated along with 25 µg/ml EVs or FPs for 1 h. Fluorescence was measured at 488 nm using a SONY SH800 flow cytometer (Sony Biotechnology) and analyzed using FlowJo version 10 software. Data is presented from at least 3 independent biological samples.

### Statistics

All data are presented as mean ± standard deviation. A One-way ANOVA was used to determine differences between multiple groups. A Tukey’s post hoc multiple comparison test was used. A *P* value < 0.05 was considered statistically significant.

## Results

### EVs and FPs were isolated and characterized from healthy and PA infected epithelial cells

EVs and FPs were isolated from cell culture supernatants using size exclusion chromatography (Fig. 1A). Each separate fraction was then characterized for EV and FP enrichment. In hTCEpi cells, the control and PA infected fractions showed no significant differences in the mean protein concentration (Fig. 1B). For HBECs, F2-F5 showed a significant drop in protein concentration in the PA infected group compared to the non-infected control. Overall, in all cell types and groups, the early fractions F1-F3 had a lower protein concentration. The highest protein concentration was detected in F4-F5. This was decreased in later fractions (F6-F7). Unlike protein concentration, there were no differences in the NTA particle numbers between control and PA infected cells of either cell type (Fig. 1C). The early fractions, F1 and F2, were more enriched with particles in the range of ∼ 1 × 10^12^ particles/mL. The particle number was decreased by 3 log units to 10^9^ for F3 in all cell types regardless of infection status. F4-F8 particle numbers were below the detection limit of < 1 × 10^6^ particles per mL for the instrument in all groups. EV purity of F1-F3 was calculated as the particle per µg of protein ratio (PP ratio). To determine the purity class for our EV fractions, we used Webber et al.’s PP ratio cutoff of 1.5 × 10^9^ particle per µg of protein, with less than that value considered to be impure with protein contamination [33]. In all test and control groups, F1 and F2 had a PP ratio above 1.5 × 10^9^ particle per µg of protein (Fig. 1D). Hence, F1 and F2 were used as our pure EV enriched fractions with minimal protein contamination. As F3 had a PP ratio below this purity range, it was more likely that along with EVs there was a substantial amount of free protein. In the later fractions, F4 and F5, the protein concentration was the highest. The PP ratio was not significantly different between control and PA infected groups in hTCEpi cells; however, it was significantly higher for PA infected HBECs compared to control cells due to a decrease in the protein concentration in PA-B fractions.

Immunoblotting was used to further characterize EV markers (Fig. 1E). F1 and F2 were positive for EV markers CD9, CD81, CD63, TSG101 and flotillin, although F2 had weaker band intensities compared to F1. The EV negative marker calnexin was absent in F1 and F2 across all groups. NTA and TEM was performed to confirm the EV size. The mean EV size in all groups ranged from 108 to 133 nm (Fig. 1F). There were no significant differences in EV size between groups. TEM of EVs from F1 and F2 pooled fractions confirmed the presence of membrane bound EV-like particles not exceeding 200 nm (Fig. 1G). Together, these data demonstrate that the early fractions, F1 and F2, are enriched with EVs, while the soluble proteins are enriched in the later fractions, F4 and F5. For all remaining experiments, pooled F1 and F2 were used as EVs and pooled F4 and F5 were used as free proteins (FPs). Since F3 seemed to be a mixed population of EVs and FPs, this fraction was excluded from further analysis.

### PA infection alters the human proteomic profile of EVs derived from corneal and bronchial epithelial cells

**Fig. 2:**
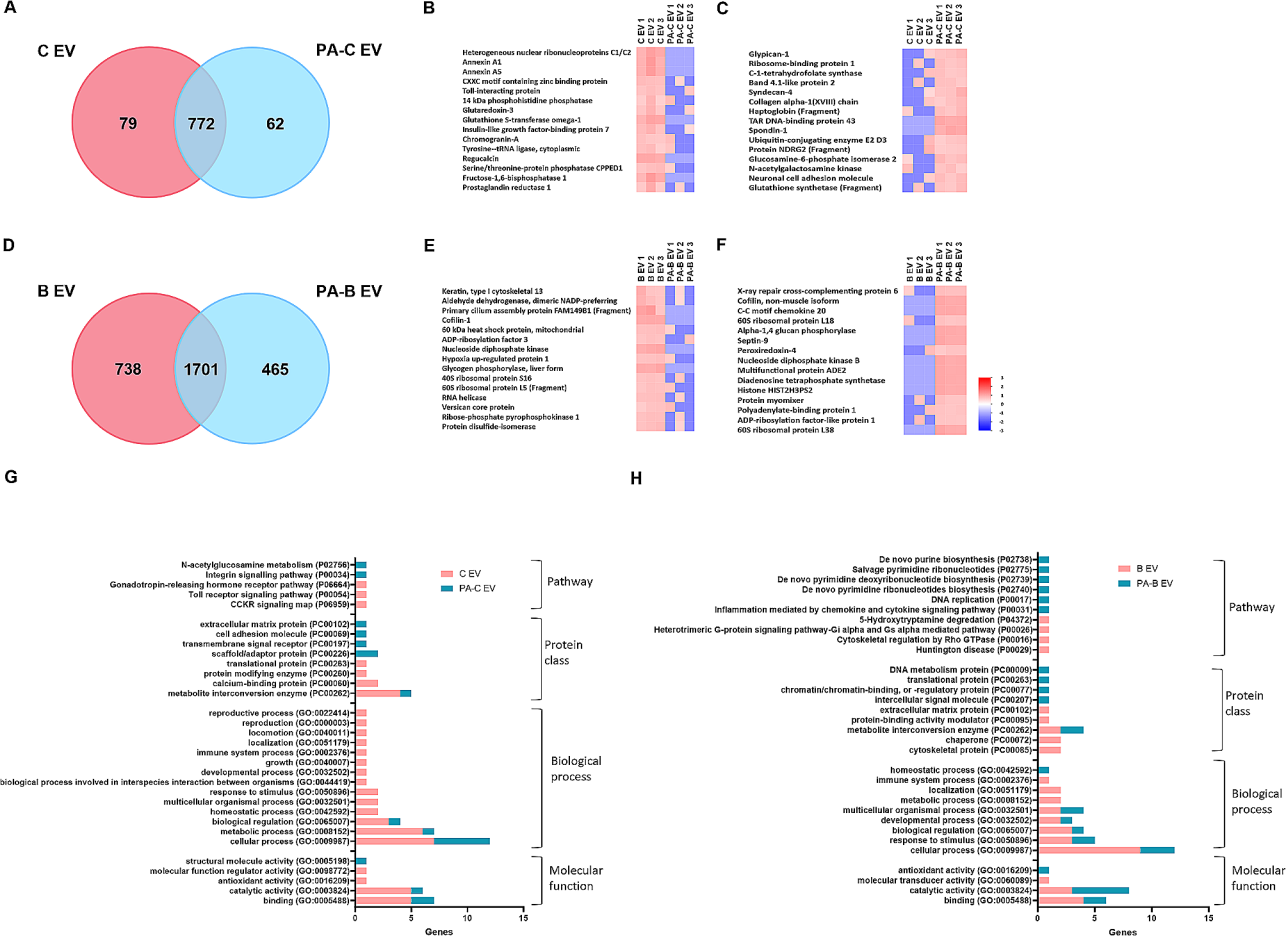
Corneal epithelial cell-derived EVs contain far fewer proteins than bronchial epithelial cell-derived EVs, and GO profiles are changing during PA infection in both cell types. Human proteomic profiles for EVs derived from non-infected and PA-infected corneal and bronchial epithelial cells. (**A**) Venn diagram showing shared and unique proteins present in EVs from hTCEpi cells; (**B**) heat map of the top 15 abundant human proteins unique to EVs from uninfected hTCEpi cells (C EVs); and, (**C**) heat map of the top 15 abundant proteins unique to EVs from PA infected hTCEpi cells (PA-C EVs). (**D**) Venn diagram showing shared and unique proteins present in EVs from HBECs; (**E**) heat map of the top 15 abundant human proteins unique to EVs from uninfected HBECs (B EVs); and, (**F**) heat map of the top 15 abundant human proteins unique to EVs from PA infected HBECs (PA-B EVs). (**G**) GO profiles comparing the unique proteins from healthy and PA infected corneal epithelial EVs. (**H**) GO profiles comparing the unique proteins from healthy and PA infected bronchial epithelial EVs. *N* = 3 biological replicates. The heat map Z score with color code (bottom right) shows the relative abundance of proteins. Red indicates higher abundance and blue indicates lower abundance. Heat map data is shown from 3 biologically independent experimental groups for each sample

We next interrogated the proteomic composition of the EV fractions collected from non-infected and PA-infected hTCEpi cells and HBECs. A total of 851 and 834 human proteins were detected in EVs collected from non-infected control (C EV) and PA-infected (PA-C EV) hTCEpi cells respectively; and 772 proteins were common to both groups (Fig. 2A). The 15 most abundant unique proteins for each group are shown as heat map in Fig. 2B. The red indicates the most abundant unique proteins and the blue indicates the lowest abundant unique proteins in the group. The 15 most abundant unique proteins in the C EV group included proteins related to cell cycle control such as heterogeneous nuclear ribonucleoproteins C1/C2, followed by proteins involved in maintaining anti-inflammatory effects and cell signaling such as annexin A1 and A2, and toll interacting proteins. (Fig. 2B). The 15 most abundant unique proteins in PA-C EV group included proteins involved in regulating cell proliferation and migration such as glypican-1, and enzymes related to cellular metabolic pathways such as C1-tetrahydrofolate synthase, glucosamine 6 phosphate isomerase, and N-acetylgalactosamine kinase (Fig. 2C). In EVs derived from HBECs, there were 2439 and 2166 proteins detected in the non-infected control (B EV) and PA-infected groups (PA-B EV), respectively (Fig. 2D). 1701 proteins were common to both groups. The 15 most abundant unique proteins in the B EV group included cytoskeletal proteins, metabolic enzymes such as aldehyde dehydrogenase, and ribosomal proteins (Fig. 2E). The top 15 unique proteins from the PA-B EV group included proteins related to DNA repair such as x-ray repair cross-complementing protein 6, immune cell chemotactic protein CCL20, carbohydrate metabolic enzymes such as alpha-1 and 4 glucan phosphorylase, and GTPases such as septin- 9 (Fig. 2F).

PANTHER gene ontology (GO) analysis was performed for the 15 most abundant unique human proteins identified in C EVs and B EVs in PA infected and non-infected cells. The GO categories identified included molecular function, which is the activity or action of a gene product or compound; biological process, which is the execution of a genetically-encoded program; protein class, which includes the commonly used class of protein families and some that are not covered under GO molecular function; and pathway [34, 35]. Genes not characterized within these 4 categories were excluded. The C EV group showed more GO categories compared to the PA-C EV group (Fig. 2G). The top GO molecular function in C EV and PA-C EV groups was binding, and for GO biological process was cellular process. There was a greater number of functions detected in the GO biological process for C EV than PA-C EV, and the total gene hits were the least for PA-C EV. For GO protein class in C EV, the top gene hit (the greatest number of genes within the category) was for metabolite interconversion enzyme, and for PA-C EV, the top hit was for scaffold/adaptor protein. Overall, both groups had unique hits for protein class, with the exception of metabolite interconversion enzyme that was present in both. In contrast, all hits in the GO pathway profile were unique between infected and non-infected groups. CCKR signaling, toll-receptor signaling and gonadotrophin-releasing hormone pathways were detected in C EV, and integrin signaling and N-acetylglucosamine metabolism were detected in PA-C EV.

Infected and non-infected B EVs had similar GO molecular function profiles with binding as the top hit for B EV and catalytic activity as top hit for PA-B EV (Fig. 2H). B EV and PA-B EV also shared similar GO biological process profiles with cellular process as the top hit. The majority of the GO protein class and GO pathways were different between B EV and PA-B EV. Overall, more human proteins were detected in B EVs than C EVs (Fig. 2A and D). PA-B EV pathway categories were tripled compared to PA-C EVs with no shared categories.

In sum, the shift in protein abundance across GO functions indicates that EV profiles are altered during PA infection. Elucidating the differences in the GO profiles of EVs derived from non-infected, healthy control cells compared to PA infected host epithelial cells provides key insights into the potential functional role of epithelial cell derived EVs during infection. In addition, the large disparity in the number of total proteins identified in EVs derived from the different cell types highlights potential differences in the underlying pathophysiology of disease.

### The host-derived FP profile suggests alterations in metabolism during PA invasion into corneal and bronchial epithelial cells

**Fig. 3:**
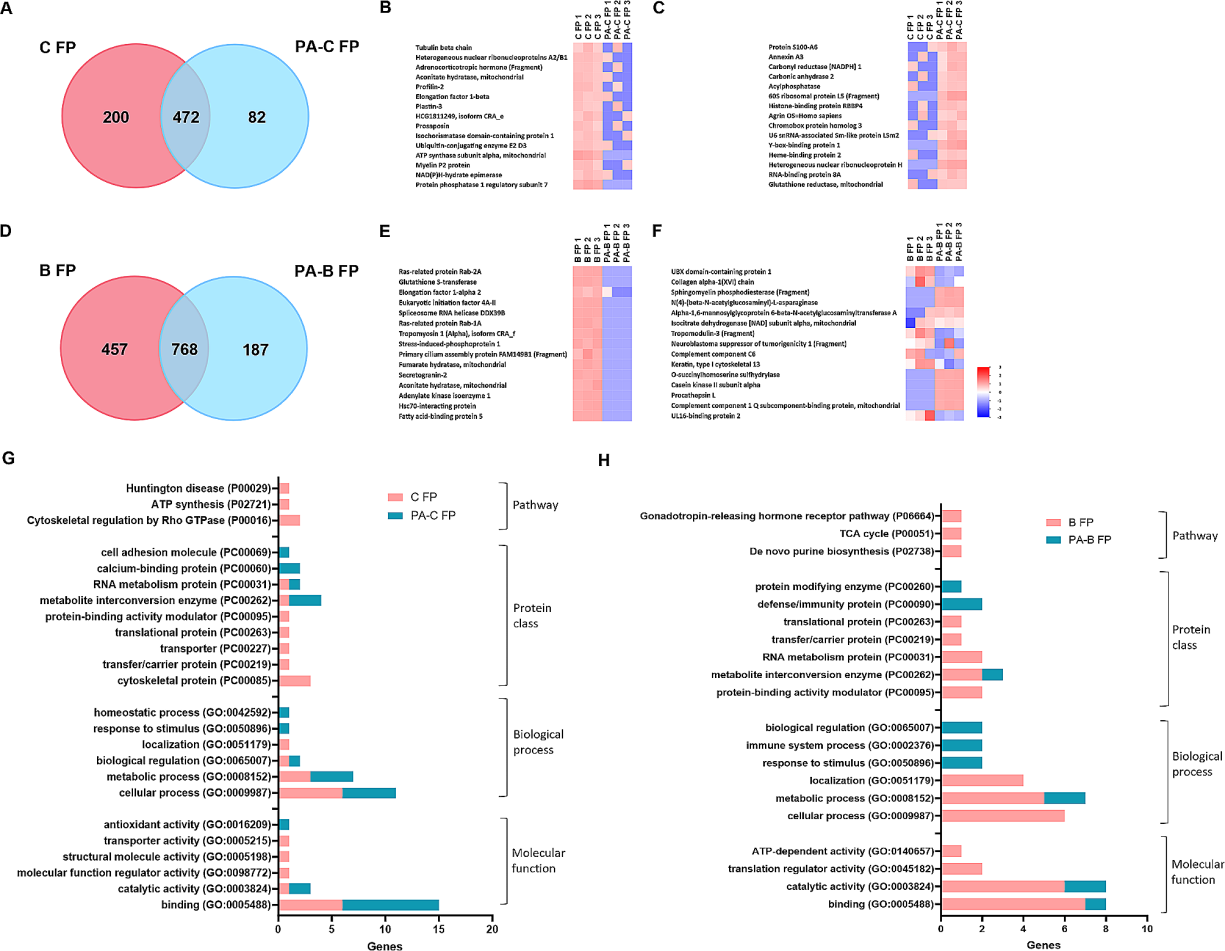
Human proteomic profiles of corneal and bronchial epithelial cell-derived FPs and GO functions are changing during PA infection, with fewer unique proteins released by infected cells. Human proteomic profiles for FPs derived from non-infected and PA infected corneal and bronchial epithelial cells. (**A**) Venn diagram showing shared and unique proteins present in FPs from hTCEpi cells; (**B**) heat map of the top 15 abundant human proteins unique to FPs from uninfected hTCEpi cells (C FPs); and, (**C**) heat map of the top 15 abundant proteins unique to FPs from PA infected hTCEpi cells (PA-C FPs). (**D**) Venn diagram showing shared and unique proteins present in FPs from HBECs; (**E**) heat map of the top 15 abundant human proteins unique to FPs from uninfected HBECs (B FPs); and, (**F**) heat map of the top 15 abundant human proteins unique to FPs from PA infected HBECs (PA-B FPs). (**G**) GO profiles comparing the unique proteins in FPs from healthy and PA infected corneal epithelial cells. (**H**) GO profiles comparing the unique proteins in FPs from healthy and PA infected bronchial epithelial cells. *N* = 3 biological replicates. The heat map Z score with color code (bottom right) shows the relative abundance of proteins. Red indicates higher abundance and blue indicates lower abundance. Heat map data is shown from 3 biologically independent experimental groups for each sample

The soluble FPs derived from the host cell, also known as the host epithelial cell secretome, provides critical information on the molecular changes associated with infection. A prior study has shown that the host epithelial cell secretome participates in immune modulation and anti-microbial responses during infection [36]. Here we analyzed the proteomic profile of FPs released by non-infected and PA infected hTCEpi cells and HBECs in order to dissect out these differences at the molecular level. Proteomic analysis detected 672 and 554 total proteins from uninfected hTCEpi cells (C FP) and PA infected (PA-C FP) FPs, respectively, with 472 proteins common for both groups (Fig. 3A). The most abundant proteins in the C FP fraction included metabolic enzymes such as aconitate hydratase, the NAD(P)HX repair enzyme NADPH hydrate epimerase, and heterogeneous nuclear ribonucleoproteins (Fig. 3B). The most abundant proteins in the PA-C FP included calcium binding protein S-100 A6, annexin A3, and metabolic pathway enzymes such as carbonyl reductase (NADPH) 1 and carbonic anhydrase 2, glutathione reductase (Fig. 3C). In FP-derived from HBECs, there were a total of 1225 and 955 human proteins detected in the B FP and PA-B FP groups, respectively, with 768 common proteins (Fig. 3D). The most abundant proteins in the B FP group included the cellular detoxification enzyme glutathione S-transferase, the chaperone adaptor protein stress-induced phosphoprotein 1, and metabolite enzymes such as fumarate hydratase and aconitate hydratase (Fig. 3E). The most abundant proteins in the PA-B FP group included the sphingolipid metabolism enzyme sphingomyelin phosphodiesterase, the N-acetylyglucosamine metabolism enzyme N [4]-(beta-N-acetylglucosaminyl)-L-asparaginase, isocitrate dehydrogenase, and procathepsin L (Fig. 3F).

Next, PANTHER GO functions were assessed for the 15 most abundant proteins detected in each group. For GO molecular function, binding was the top hit for both C FP and PA-C FP. There were more categories detected in C FP than PA-C FP (Fig. 3G). For GO biological process, cellular process and metabolic process were the top 2 hits for both groups. For GO protein class, while there were many categories unique to each group, C FP and PA-C FP shared RNA metabolism and metabolite interconversion categories (Fig. 3G). GO pathways such as Huntington disease, ATP synthesis and cytoskeletal regulation of Rho GTPase were unique to C FP with no pathway category detected in PA-C FP (Fig. 3G).B FP and PA-B FP shared fewer than half of the GO categories, and most differed across groups (Fig. 3H). The GO molecular function top hit was binding for B FP and catalytic activity for PA-B FP. The GO biological process top hit was cellular process for B FP, while response to stimulus, immune system process and biological regulation were exclusive to PA-B FP. Top categories for GO protein class were protein-binding activity modulator, metabolite interconversion enzyme and RNA metabolism protein for B FP, whereas defense/immunity protein was highest for PA-B FP. Finally, GO pathway categories in B FP included *de novo* purine synthesis and TCA cycle. There were no GO pathway hits for PA-B FP. Overall, for both hTCEpi cells and HBECs, GO PANTHER gene hits were less for FPs derived from PA infected cells compared to uninfected controls. Importantly, the FP proteomic profiles were changing during infection in both cell types, indicative of functional changes in cell homeostasis and metabolism.

### Bacterial-derived proteins present in EVs and FPs derived from PA-infected corneal and bronchial epithelial cells exhibit a unique signature

**Fig. 4:**
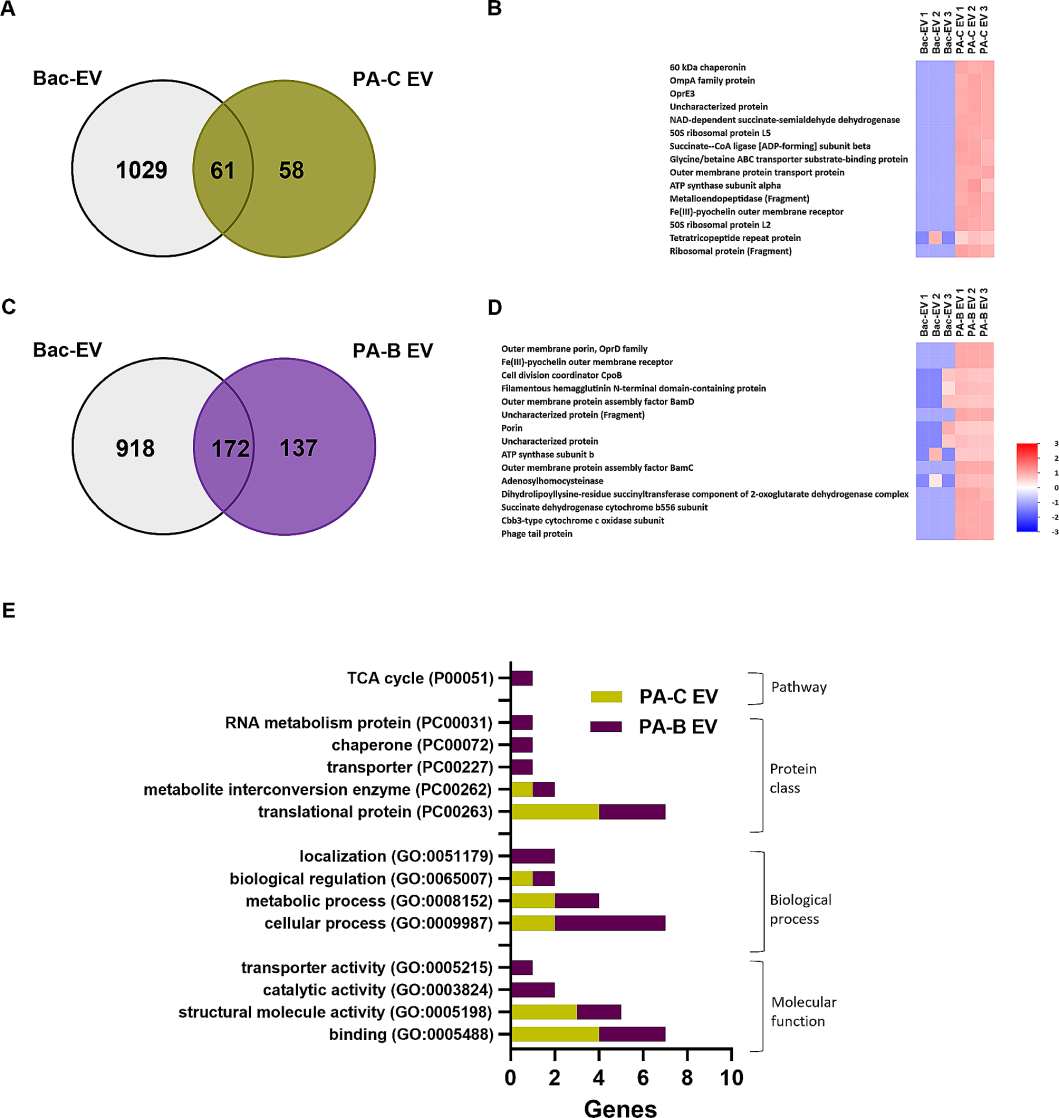
The bacterial proteomic profiles of PA-infected host epithelial cell derived EVs compared to bacterial-derived EVs show differences during infection. (**A**) Venn diagram showing shared and unique proteins present in bacterial-derived EVs (Bac EVs) and EVs derived from PA-infected hTCEpi cells (PA-C EVs); and, (**B**) heat map of the top 15 bacterial proteins unique to PA-C EVs. (**C**) Venn diagram showing shared and unique proteins present in Bac EVs and EVs derived from PA-infected HBECs (PA-B EVs); and, (**D**) heat map of the top 15 bacterial proteins unique to PA-B EVs. (**E**) GO profiles of unique bacterial proteins detected in PA infected corneal and bronchial epithelial cell derived EVs (PA-C EV and PA-B EV). *N* = 3 biological replicates. Heat map Z score with color code (bottom right) shows the relative abundance of proteins. Red indicates higher abundance and blue indicates lower abundance. Heat map data is shown from 3 biologically independent experimental groups for each sample

We next investigated differences in the profile of bacterial proteins in PA-C EVs, PA-B EVs and the associated FPs during infection. To better understand the function of the bacterial proteins unique to EVs from infected host cells, we compared PA-C EVs and PA-B EVs with bacterial EVs from planktonic PA [12]. A total of 119 bacterial proteins were detected in PA-C EVs, 58 of which were unique (Fig. 4A). The top 15 most abundant proteins from these 58 unique proteins were plotted as a heat map. These included outer membrane proteins OmpA family protein, and OprE3 (Fig. 4B). In PA-B EVs, a total of 309 proteins were identified (Fig. 4C), with 137 being unique to this EV group. The top 15 from this list are shown in Fig. 4D. These include outer membrane proteins OprD, porin, and BamD. PANTHER GO analysis was performed for the bacterial proteins unique to PA-C EVs and PA-B EVs. Consistent with fewer unique proteins being present in PA-C EVs compared PA-B EVs, there were fewer GO categories detected in PA-C EVs compared to PA-B EVs (Fig. 4E). RNA metabolism protein, chaperone, transporter, and TCA cycle were exclusively found in PA-B EVs (Fig. 4E).

**Fig. 5:**
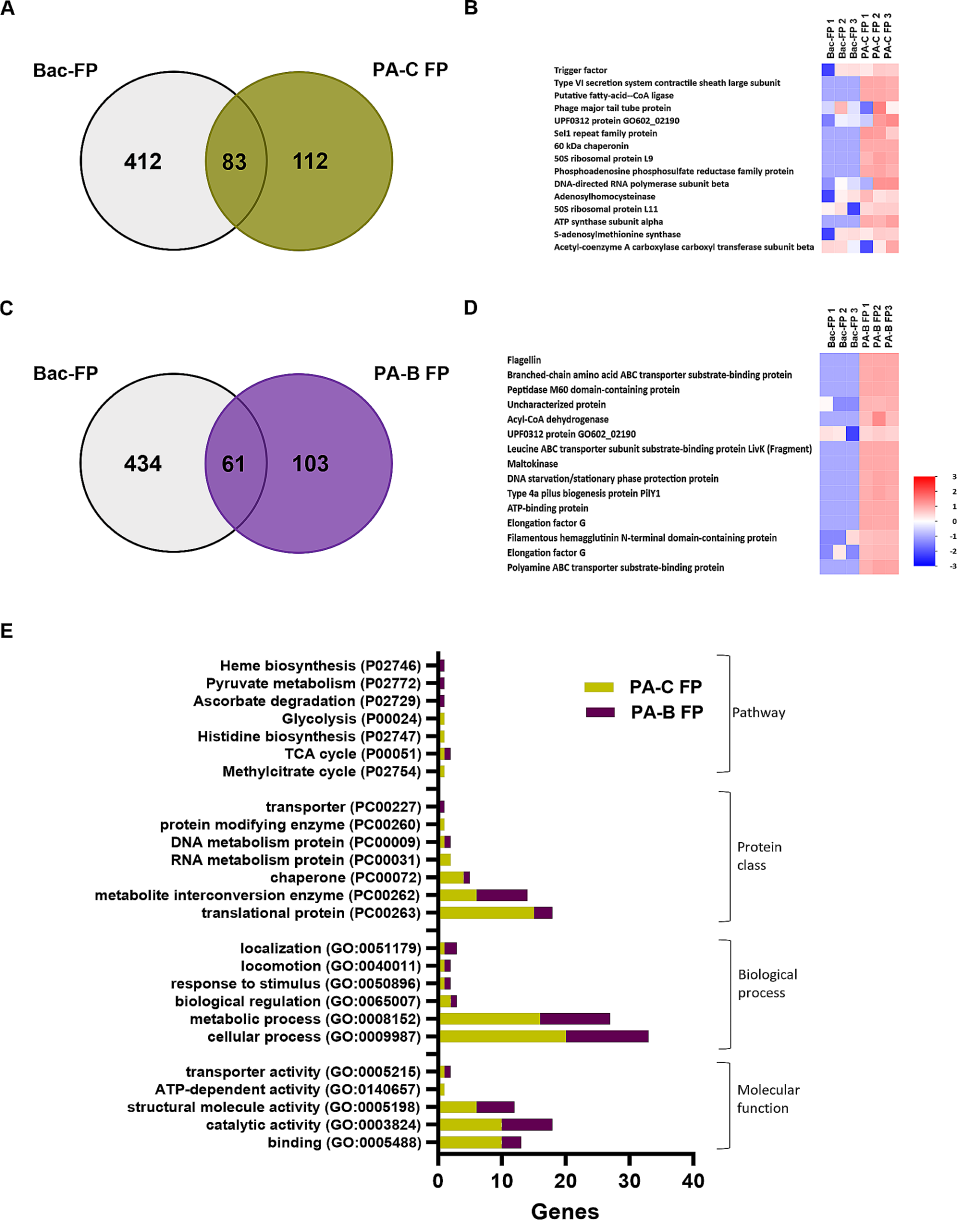
PA infected host epithelial cell derived FPs have unique bacterial proteins compared to bacterial-derived FPs. (**A**) Venn diagram showing shared and unique proteins present in bacterial-derived FPs (Bac FPs) and FPs derived from PA infected hTCEpi cells (PA-C FPs); and, (**B**) heat map of the top 15 bacterial proteins unique to PA-C FPs. (**C**) Venn diagram showing shared and unique proteins present in Bac FPs and FPs derived from PA-infected HBECs (PA-B FPs); and, (**D**) heat map of the top 15 bacterial proteins unique to PA-B FPs. (**E**) GO profiles of unique bacterial proteins detected in PA infected corneal and bronchial epithelial cell derived FPs (PA-C FP and PA-B FP). *N* = 3 biological replicates. Heat map Z score with color code (bottom right) shows the relative abundance of proteins. Red indicates higher abundance and blue indicates lower abundance. Heat map data is shown from 3 biologically independent experimental groups for each sample

Similarly, in PA-C FP, a total of 195 bacterial proteins were detected during infection, with 112 being unique to PA-C FP when compared to planktonic culture (Fig. 5A). Again, the top 15 most abundant proteins unique to PA-C FP were plotted as heat map (Fig. 5B). Since the average of log2 abundance from 3 independent samples was used for calculating the most abundant proteins for the heat map, trigger factor protein was plotted as the topmost abundant protein in the group for Bac-FP compared to PA-C FP. However, it should be noted that these proteins were detected in 2 out of 3 samples from the Bac-FP group and at similar amounts in the PA-CFP group. The other 2 topmost abundant bacterial proteins in the PA-C FP group included Type VI secretion system proteins and putative fatty-acid-CoA ligase. In PA-B FP, a total of 164 bacterial proteins were present, with 103 unique proteins (Fig. 5C, top 15 in Fig. 5D). PANTHER GO analysis was performed for the bacterial proteins unique to PA-C FPs and PA-B FPs. Most of the GO functions, except GO pathway, were similar in both PA-C FP and PA-B FP (Fig. 5E). In sum, bacterial proteins detected in EVs derived from hTCEpi cells and HBECs during PA infection exhibited unique profiles with respect to cell type and GO functions. In contrast, FPs released by each cell type shared similar GO functions for most categories. EVs derived from both cell types were enriched with various bacterial outer membrane proteins which may impact innate immune responses, while FPs were enriched with various bacterial metabolic proteins and enzymes.

### Innate immune responses in corneal and bronchial epithelial cells are regulated by PA-derived EVs and FPs

**Fig. 6:**
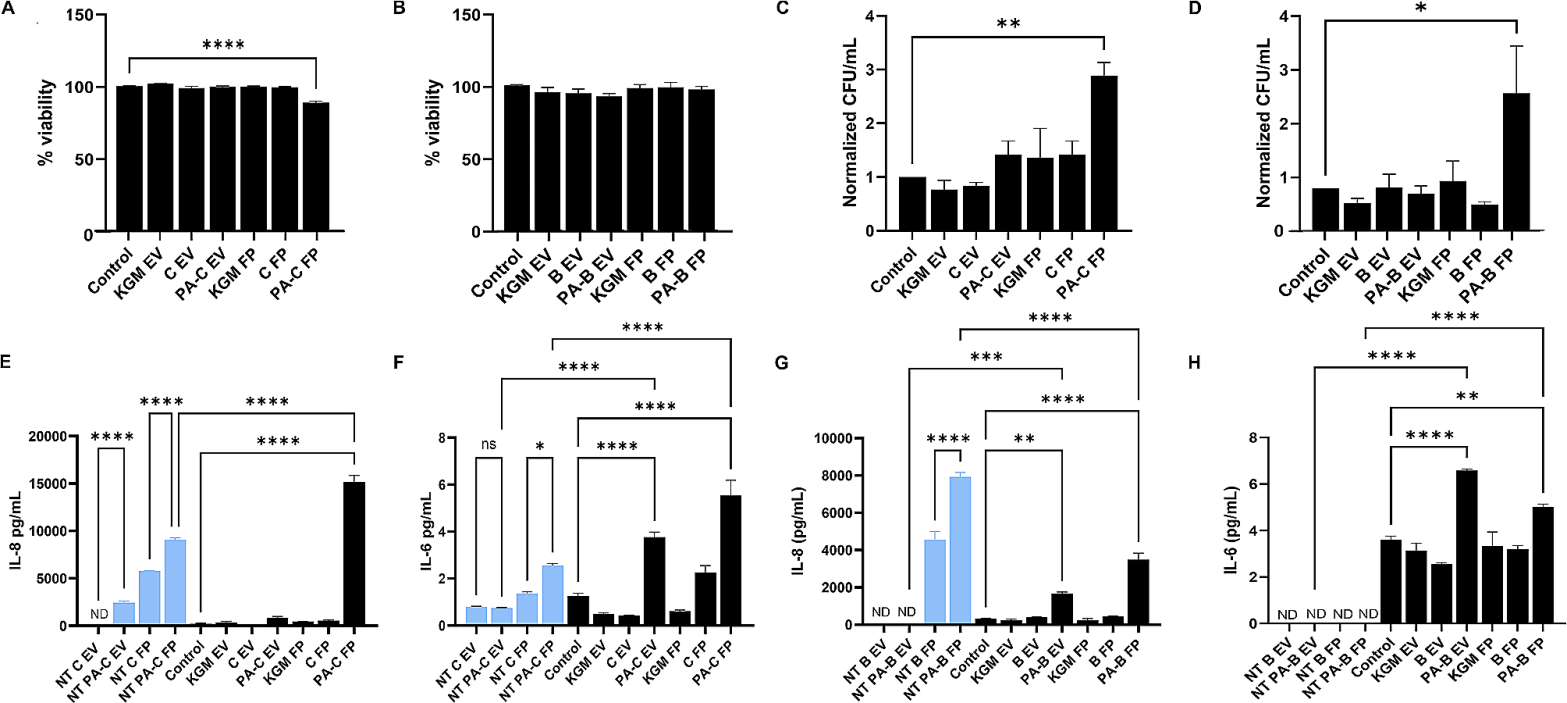
EVs and FPs released by PA-infected epithelial cells exert distinct responses in naïve, non-infected epithelial cells. Naïve, non-infected cells were treated with EVs and FPs released by healthy and PA-infected cells. The naïve, non-infected cells without any treatment were included as a control and the EV and FP fractions isolated from growth medium (KGM) with hTCEpi cells and HBECs served as an additional control (indicated as KGM EV and KGM FP). (A-B) With the exception of PA-C FP, none of the treatments reduced viability in (**A**) hTCEpi cells or (**B**) HBECs. (**C**-**D**) A gentamicin survival assay was performed to detect intracellular PA survival. PA infected host derived EVs had no effect on intracellular PA survival. In contrast, treatment with FPs increased intracellular levels of PA in both (**C**) hTCEpi cells and (**D**) HBECs. (E-H) ELISA was performed to detect pro-inflammatory cytokine secretion. Levels of IL-6 and IL-8 in EVs and FPs isolated from the host cells were first measured (indicated by NT). Then levels of IL-6 and IL-8 secretion by non-infected cells treated with either EVs or FPs were quantified. (**E**) Treatment with PA infected host-derived FPs increased IL-8 secretion in corneal epithelial cells. (F) Treatment with PA infected host-derived EVs and FPs also increased IL-6 secretion in corneal epithelial cells. (G-H) Treatment with PA infected host-derived EVs and FPs led to higher levels of IL-8 (**G**) and IL-6 (**H**) in bronchial epithelial cells compared to the no treatment control, although IL-8 levels for PA-B FP group was lower than levels present in NT PA-B FPs. This indicates that PA-B FPs did not induce secretion of IL-8 in bronchial epithelial cells. Data presented as mean ± standard deviation. Graphs representative of 3 independent experiments performed in triplicate. One way ANOVA with Tukey post hoc multiple comparison test, *p < 0.05, **p < 0.01, ***p < 0.001, ****p < 0.0001

We next investigated the effects of EVs derived from PA infected hTCEpi cells and HBECs on naïve, non-infected epithelial cells. To accomplish this, we first determined whether EVs derived from PA infected cells exhibited any level of cytotoxicity. In all experiments, cells were treated with 25 µg/mL of EVs or FPs isolated from PA-infected epithelial cells of the corresponding cell type. Due to the protein concentrations for EVs and the FPs isolated from the growth media control (no cells) being below 25 µg/mL, in order to treat the naïve cells, we used a volume that was equivalent to the lowest protein concentration of EVs or FPs derived from our infected samples for the control groups. Cytotoxicity was measured using an LDH assay. After 24 h of treatment, there was no change in the viability of any of the test or control epithelial cells, with the exception of PA-C FP (Fig. 6A-B). PA-C-FP caused a small decrease in the viability of corneal epithelial cells. This may be due to the presence of acylphosphatase which has been shown to cause apoptosis in HeLa [37]. 

It is well established that PA is an intracellular pathogen that can survive and replicate inside of epithelial cells [38, 39]. These processes are mediated in part by virulence factors [7, 40–42]. To determine whether EVs released by PA-infected host cells conferred a protective or deleterious effect on PA invasion of hTCEpi cells or HBECs, we next performed a gentamicin invasion assay. While we were unable to demonstrate any effect of PA-C EVs or PA-B EVs on intracellular PA survival (Fig. 6C-D), treatment with PA-C FPs and PA-B FPs increased the intracellular bacterial load in their respective cell types (Fig. 6C-D).

To determine the effects of EVs and FPs derived from PA infected cells on subsequent innate immune responses of naive, non-infected epithelial cells, we examined pro-inflammatory cytokine secretion. To do this, non-infected hTCEpi cells and HBECs were treated with 25 µg/mL of EVs or FPs isolated from their respective cell types for 24 h. Levels of IL-8 and IL-6 in cell culture supernatants were measured by ELISA. Baseline levels of cytokines in the experimental EVs and FPs were tested prior to addition to non-infected cells (referred to as no treatment (NT) groups). IL-8 was detected inside the NT PA-C EVs and was significantly higher in NT PA-C FPs (Fig. 6E). Treatment of non-infected hTCEpi cells with PA-C FPs increased IL-8 secretion to levels much higher than levels present in NT PA-C FPs, indicating active secretion by non-infected hTCEpi cells (Fig. 6E). PA-C EVs failed to alter secretion of IL-8 in non-infected cells. Low levels of IL-6 were detected in NT PA-C EVs but at levels similar to NT C EVs. IL-6 was enriched in NT PA-C FP compared to controls. However, treatment with either PA-C EVs or PA-C FPs significantly increased IL-6 secretion in non-infected hTCEpi cells, again indicating cellular secretion upon stimulation (Fig. 6F).

In HBECs, neither IL-8 nor IL-6 was present in NT PA-B EVs (Fig. 6G-H). IL-8, but not IL-6, was present in NT PA-B FPs. Treatment of non-infected HBECs with PA-B EVs increased both IL-8 and IL-6. Secretion of IL-8 and IL-6 was similarly increased by treatment with PA-B FP. Since there was no detectable level of IL-8 or IL-6 inside the NT PA-B EVs, these data indicates that HBECs secrete both cytokines upon stimulation with PA-B EVs. This response was similar for IL-6 secretion in response to treatment with PA-B FPs. In contrast to these findings, IL-8 was not increased by treatment with PA-B FP, despite high levels of IL-8 in the NT PA-B FPs.

Taken together, these findings show that EVs and FPs released by PA infected epithelial cells are capable of inducing pro-inflammatory cytokine secretion in non-infected epithelial cells, albeit with differential responses between cells type. While the increased levels of IL-8 corresponded to an increase in intracellular levels of PA in hTCEpi cells, this response was not evident in HBECs. Further interrogation into the specific components present in PA-C FP and PA-B FP are needed to determine the mechanism underlying the increase in intracellular PA.

### Corneal and bronchial epithelial cell derived EVs regulate neutrophil function during PA infection

**Fig. 7:**
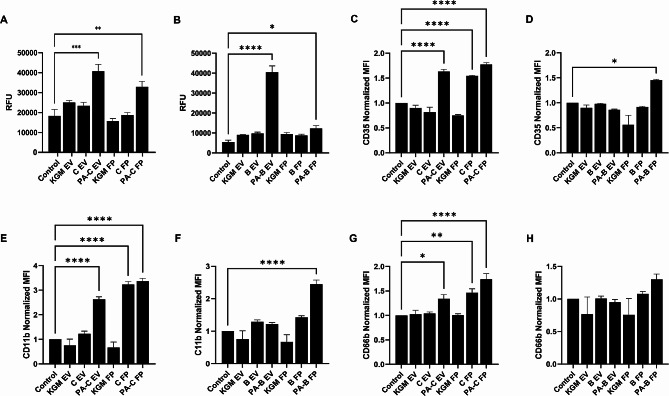
EVs released by PA infected epithelial cells are chemotactic for neutrophils. The naïve, non-infected cells without any treatment were included as a control and the EV and FP fractions isolated from growth medium (KGM) with hTCEpi cells and HBECs served as an additional control (indicated as KGM EV and KGM FP). (A-B) Neutrophil migration was measured using a Boyden-chamber assay (RFU, relative fluorescence intensity). (**A**) EVs released by PA infected hTCEpi cells and FPs increased neutrophil migration; and, (**B**) EVs derived from PA infected HBECs similarly increased neutrophil migration. (C-H) Neutrophil activation markers CD35, CD66b and CD11b expression shown as normalized mean fluorescence intensity (MFI) following treatment with EVs and FPs derived from (**C**, **D**, **E**) hTCEpi cells, and (**F**, **G**, **H**) HBECs. EVs and FPs from PA infected hTCEpi cells induced expression of neutrophil activation markers. Data presented as mean ± standard deviation. Graphs representative of 3 independent experiments performed in triplicate. One way ANOVA with Tukey post hoc multiple comparison test, *p < 0.05, **p < 0.01, ***p < 0.001, ****p < 0.0001

Neutrophils are the first line of innate immune responders during corneal and airway infections [43–46]. Neutrophils are recruited to the site of infection and function to eliminate pathogens through their bactericidal activity [44, 47]. To determine the effects of PA-derived EVs and FPs on neutrophils, we first examined their chemotactic potential using a standard Boyden chamber assay. Importantly, using differentiated HL-60 cells and peripheral blood neutrophils, we found that both PA-C EVs and PA-B EVs induced neutrophil migration (Fig. 7A-B, Supplementary Fig. 1A-B). PA-C FP was also chemotactic to neutrophils, whereas PA-B FP was not. We further looked into other neutrophil functions such as respiratory burst and bacteria killing. Neither PA-C EVs nor PA-B EVs altered respiratory burst (Supplementary Fig. 1C-D) or bacterial killing (Supplementary Fig. 1E-F). Finally, we examined the expression levels of neutrophil activation markers CD35, CD66b and CD11b by flow cytometry [48]. Supporting Fig. 1G shows the flow cytometry gating strategy that was used. We found that PA-C EVs and PA-C FPs significantly increased CD35, CD66b and CD11b expression indicating neutrophil activation compared to the no treatment control (Fig. 7C, E, & G). Interestingly, C FP treatment also significantly increased expression of all 3 neutrophil activation markers (Fig. 7C, E, & G ). PA-B EV treatment had no impact on the neutrophil activation marker expression, however PA-B FP treatment increased CD35 and CD11b expression (Fig. 7F, G, & H). Overall, these data show that host derived EVs and FPs from infected corneal and bronchial epithelial cells differentially modulate neutrophil chemotaxis and neutrophil activation.

## Discussion

This study investigated the role of host derived EVs and FPs from PA-infected corneal and bronchial epithelial cells in the regulation of innate immune responses. Our first key finding was the differential effect of EVs and FPs on pro-inflammatory cytokine secretion by naïve, non-infected cells. This response differed for each cytokine and between cell types. Importantly, we only detected IL-8, a major chemoattractant, inside EVs derived from PA infected corneal epithelial cells, whereas IL-8 was present in FPs from both epithelial cell types. In corneal epithelial cells however, only FPs increased secretion of IL-8 by non-infected cells, while in bronchial epithelial cells this effect was mediated by EVs. Despite EVs not being able to promote IL-8 secretion in non-infected corneal epithelial cells, EVs were still able to induce significant chemotaxis. Similarly, in bronchial epithelial cells, while both EVs and FPs increased IL-8 secretion, EVs were the primary chemotactic stimulus. Taken together, these data suggest that other cellular or pathogen-specific factors, apart from IL-8, function to amplify the chemoattractant properties of PA infected epithelial cell derived EVs.

In addition to chemotaxis, EVs and FPs derived from PA-infected corneal epithelial cells induced expression of neutrophil activation markers CD35, CD66b, and CD11b. This is consistent with a recent study showing that EVs released by swarming neutrophils are able to promote neutrophil activation via galectin-3 [49]. Similarly, EVs derived from the gastric cancer (GC) microenvironment have been shown to induce PD-L1 expression in neutrophils and this in turn downregulates T-cell immunity [50]. GC-derived EVs have also been shown to activate neutrophils via HMGB1/TLR4/NF-kB and induce autophagy [51]. In terms of neutrophil migration, EVs from another gram-negative bacteria, *Escherichia coli*, are able to promote neutrophil migration, however, this is through endothelial IL-8 induction. Opposite these studies, we found that EVs derived PA infected bronchial epithelial cells had no effect on neutrophil activation, highlighting clear functional differences between cell types.

In cystic fibrosis patients, airway derived-EVs have been shown to induce neutrophil recruitment and exhibit varied proteomic profiles that are age-dependent [52]. We similarly compared the proteomic profiles of EVs and FPs from two different cell types that exhibited differential effects on neutrophil recruitment and found unique signatures for each. Despite identical culture conditions, we further found that the complexity of the proteome in EVs and FPs derived from corneal epithelial cells was much lower compared to EVs and FPs derived from bronchial epithelial cells. Complexity aside, the overall number of proteins was reduced for both epithelial cell types during PA infection. This suggests a downregulation in gene expression at either the transcriptional or translational level. Similar to our study, human cell lines infected with coxsackievirus B3 have also demonstrated changes in the host proteome, with the majority of changes due to the down regulation of protein expression via post translational modification [53]. Further work is needed to investigate the significance of these findings.

Anti-inflammatory proteins such as annexin 1 and annexin 2 were found to be abundant in healthy corneal epithelial cell derived EVs and were notably absent in the PA infected corneal epithelial EVs. Recent studies have shown that, apart from anti-viral immunity and pathogen clearance functions [54], annexin 1 attenuates neutrophil migration and IL-6 expression through formyl peptide receptor 2 in a *Streptococcus suis* induced meningitis mouse model [55]. Annexin 2 is able to function as a receptor for PA, and soluble annexin 2 has been shown to block bacterial ligands and inhibit PA internalization [56]. Thus, the absence of these proteins in PA-C EVs may be beneficial for the pathogen’s establishment in the host and modulation of the host immune response. Similarly, the absence of toll-interacting proteins from PA-C EVs may also promote neutrophil and other immune cell recruitment, as these proteins are known to attenuate neutrophil and T cells responses in bacterial infections [57, 58]. In contrast to these proteins, glypican-1 and sundican-4 were present in PA-C EVs, both of which may explain the increase in neutrophil recruitment [59, 60]. Lastly, our unpublished data indicates that metabolic rewiring occurs in PA infected corneal epithelial cells (manuscript in review). The presence of C1 tetrahydrofolate synthase, an enzyme involved in folate metabolism, may play a regulatory role in host metabolic adaptations during PA infection [61]. 

In bronchial epithelial cells, CCL20 was a highly abundant unique protein in PA-B EVs. Lung epithelial cell derived CCL20 has been shown to be critical for neutrophil recruitment during bacterial infection [62]. Septin-9 was also abundant in PA-B EVs. Septins are cytoskeletal proteins that can form cages around cytosolic bacteria to prevent intracellular replication and target the pathogen to the autophagy pathway [63]. Thus, the targeted release of Septin-9 by infected cells may represent a mechanism whereby the pathogen manipulates the host response to evade clearance during infection.

Aconitate hydratase was found to be exclusively abundant in C FP fractions. Aconitate hydratase is a metabolic enzyme involved in the formation of the TCA cycle intermediate cis-aconitate, a known precursor of the antimicrobial metabolite, itaconate. Itaconate is known to regulate immune function during ocular infections [64, 65]. The absence of this enzyme in PA-C FP could be beneficial for pathogen survival. The absence of glutathione S-transferase from PA-B FP could also impact PA invasion in bronchial epithelial cells, in addition to exerting effects on cell signaling, immune cell function and energy metabolism [66]. In PA-C FP, the calcium binding protein S100A6, was the most abundant protein. S100A6 has known functions as a damage associated molecular pattern (DAMPs) during bacterial infection and modulates the inflammatory response [67]. Similarly, sphingomyelin phosphodiesterase was present in PA-B FP. Sphingomyelin phosphodiesterase breaks down sphingomyelin to phosphocholine and ceramide. Several pathogens are known to exploit host phosphocholine metabolism for their survival and immune evasion [68]. Ceramide also plays important functions in bacterial internalization, intracellular signaling and cytokine release during bacterial infections [69]. 

Like human proteins, bacterial proteins present in EVs and FPs were also shifted during infection. Pathway analysis showed that this included changes in proteins involved in bacterial metabolism. One of the most abundant proteins in PA-C EVs, OmpA, is involved in host innate immune signaling and cell adhesion during PA infection [70]. Changes in bacterial metabolism are critical for virulence and survival in the host [71]. In addition, intracellular pathogens such as *Legionella*, *Coxiella*, *Listeria* and *Chlamydia* have all been shown to possess a bipartite metabolism which allows them to temporally and spatially modulate host metabolic activity [72]. Consistent with our findings of changes in bacterial metabolic proteins, PA isolates harvested from the cystic fibrosis airway demonstrated metabolic rewiring and increased expression of metabolic pathway enzymes related genes [73, 74]. The abundance of OprD and porin in PA-B EVs may also have a functional role in mediating antibiotic resistance through circulating EVs in the lung epithelium [75]. 

Other studies have primarily focused on the immunogenic potential of bacterial proteins inside host EVs [76–79]. For example, *Mycobacterium avium* is able to induce proinflammatory responses through their glycopeptidolipids that activate toll-like receptor ligands in macrophages [76]. Intranasal injection of exosomes from *Mycobacterium tuberculosis* and *Mycobacterium bovis* infected macrophages stimulate TNFα and IL-12 production, along with neutrophil and macrophage recruitment into the murine lung [78]. Similar to this prior work, EVs derived from PA infected epithelial cells exerted immunogenic effects and induced neutrophil chemotaxis. Given the widespread upregulation of IL-6 and IL-8 across EVs and FPs in this study, this may be due to the presence of bacterial proteins present in each. We previously demonstrated the ability of bacterial-derived EVs from PA to increase IL-6 and IL-8 as well as induce neutrophil migration and respiratory burst. Thus, one mechanism that may account for the current findings is the presence of a mixed population of bacterial and host-derived EVs. This is not supported however, by the observation that EVs released from both corneal and bronchial epithelial cells failed to induce respiratory burst, unlike bacterial-derived EVs. This suggests that this effect is mediated by specific factors present within host-derived EVs. Further exploration of the molecular pathways by which host derived EVs and FPs exert their regulatory effect on innate immune responses are needed.

Limitations to the current study include the absence of an animal model and investigation into the effect of the EV isolation protocol on the molecular composition of EVs. Here we chose to use size exclusion chromatography to prevent the potential rupture of EVs during centrifugation. Given the heterogenous nature of EVs, it is possible that different isolation methods may yield differences in the molecular composition. Despite these limitations, this is the first study to define the molecular signature of host derived EVs and FPs and their associated functional effects on innate immune cells during PA infection of corneal and bronchial epithelial cells.

Lastly, exosomes released by viral infected cells have been shown to exhibit cytotoxicity. HIV infected cells export a viral accessory protein known as Nef through exosomes to induce T cell cytotoxicity, a key contributory step in the pathogenesis of AIDS [80]. Nef has also been shown to induce cytotoxicity in non-infected bystander cells [81]. In the case of PA infection, host-derived EVs did not induce any measurable level of cytotoxicity. Host-derived FPs however, did increase the intracellular survival of PA. This effect was independent of cell type. Mechanistically, it is unclear how FPs exerted this effect. EVs released from *Mycobacterium tuberculosis* infected neutrophils have been shown to decrease intracellular bacterial load due to an increase in macrophage autophagy [82]. Similarly, PA has been shown to induce autophagy in alveolar macrophages and treatment with the autophagy inducer rapamycin further increased bacterial clearance in that cell type [83]. The role of autophagy in mediating intracellular clearance of PA in corneal and bronchial epithelial cells however, appears to differ. In the lung, exotoxins from the type 3 secretion system have been shown to inhibit autophagy [84]. In contrast to this, our own unpublished data (manuscript in review) demonstrates that PA induces robust autophagy in corneal epithelial cells and that perturbation of autophagy flux through activation or inhibition reduces intracellular levels of PA. Since FPs were able to increase intracellular levels of PA in both cell types, it argues against autophagy as the primary underlying mechanism. Additional studies are now underway.

In summary, PA-infection results in a change in the proteomic composition of host-derived EVs and FPs. As a large amount of these proteins were classed as metabolism related proteins, future studies are needed to investigate the ability of PA to induce host metabolic reprogramming during infection. In addition to proteomic changes, EVs derived from PA infected non-immune host cells are capable of evoking innate immune responses by amplifying neutrophil recruitment. Despite this amplification, there was not a corresponding increase in respiratory burst or neutrophil-mediated bacterial killing. This, coupled with our prior work showing that PA-derived EVs are able to impair neutrophil bactericidal activity suggests the presence of a tug-of-war between host cell and pathogen whereby the host cell EVs promote recruitment of neutrophils to the site of infection while pathogen derived EVs inactivate them. The ability of the pathogen to overwhelm the host response in the absence of bacterial clearance may play a major role in the dysregulated neutrophil response that contributes to the severe disease phenotype seen in corneal and airway disease.

### Electronic supplementary material

Below is the link to the electronic supplementary material.

Supplementary Material 1

Supplementary Material 2

## Data Availability

The proteomic dataset is available in the following database: MassIVE Repository, MSV000090310.
