# Supplementary figures and images for: Extracellular vesicles released by host epithelial cells during Pseudomonas aeruginosa infection function as homing beacons for neutrophils
Source: Cell Commun Signal. 2024 Jun 21;22:341. doi: 10.1186/s12964-024-01609-7 (PMC11191230; doi:10.1186/s12964-024-01609-7)

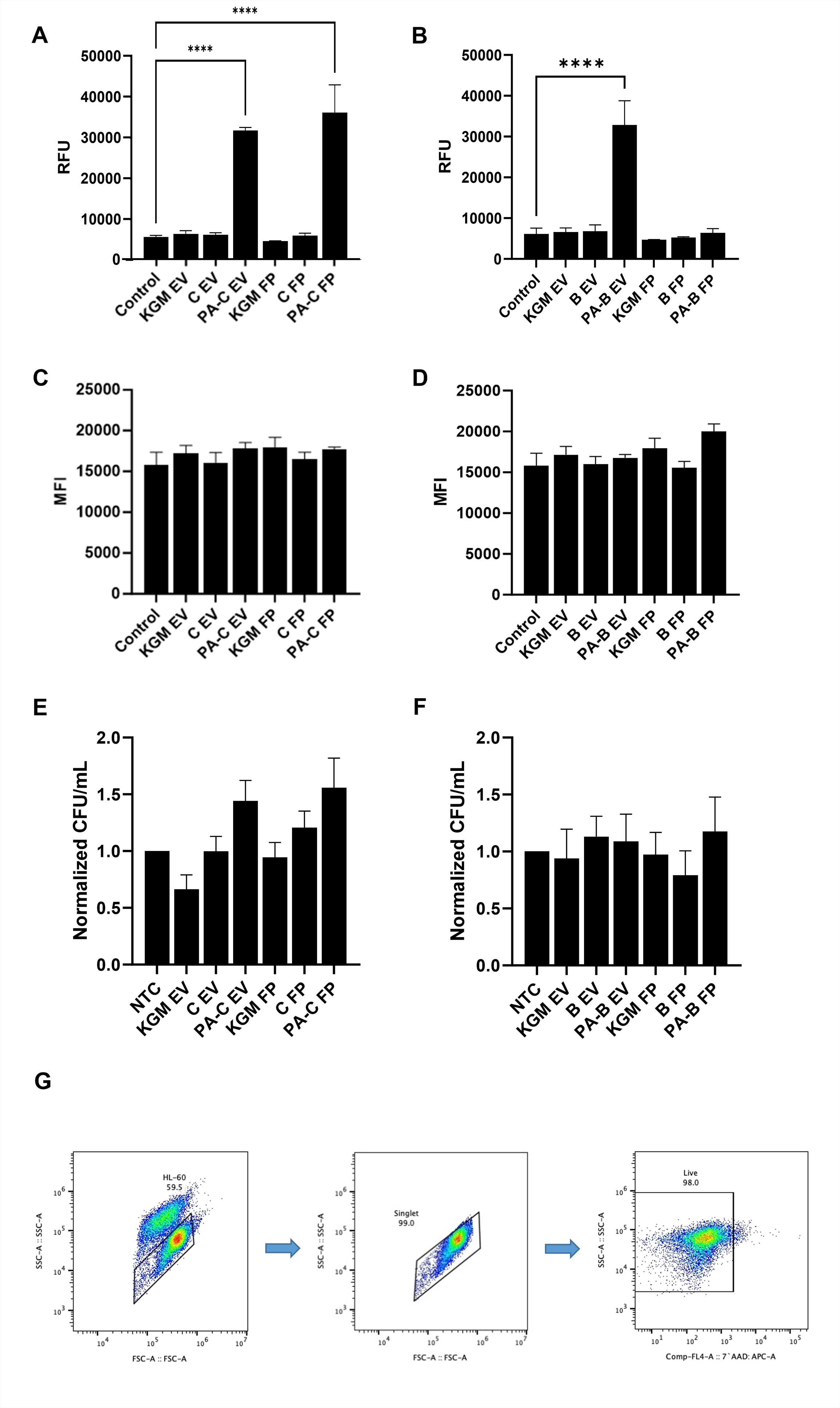

Supplement: Supplementary file 1 — Supplementary Material 1 [file 12964_2024_1609_MOESM1_ESM.png]
