# Supplementary material for: Extracellular vesicles released by host epithelial cells during Pseudomonas aeruginosa infection function as homing beacons for neutrophils
Source: Cell Commun Signal. 2024 Jun 21;22:341. doi: 10.1186/s12964-024-01609-7 (PMC11191230; doi:10.1186/s12964-024-01609-7)
Supplement: Supplementary file 2 — Supplementary Material 2 [file 12964_2024_1609_MOESM2_ESM.pptx]

## Slide 1
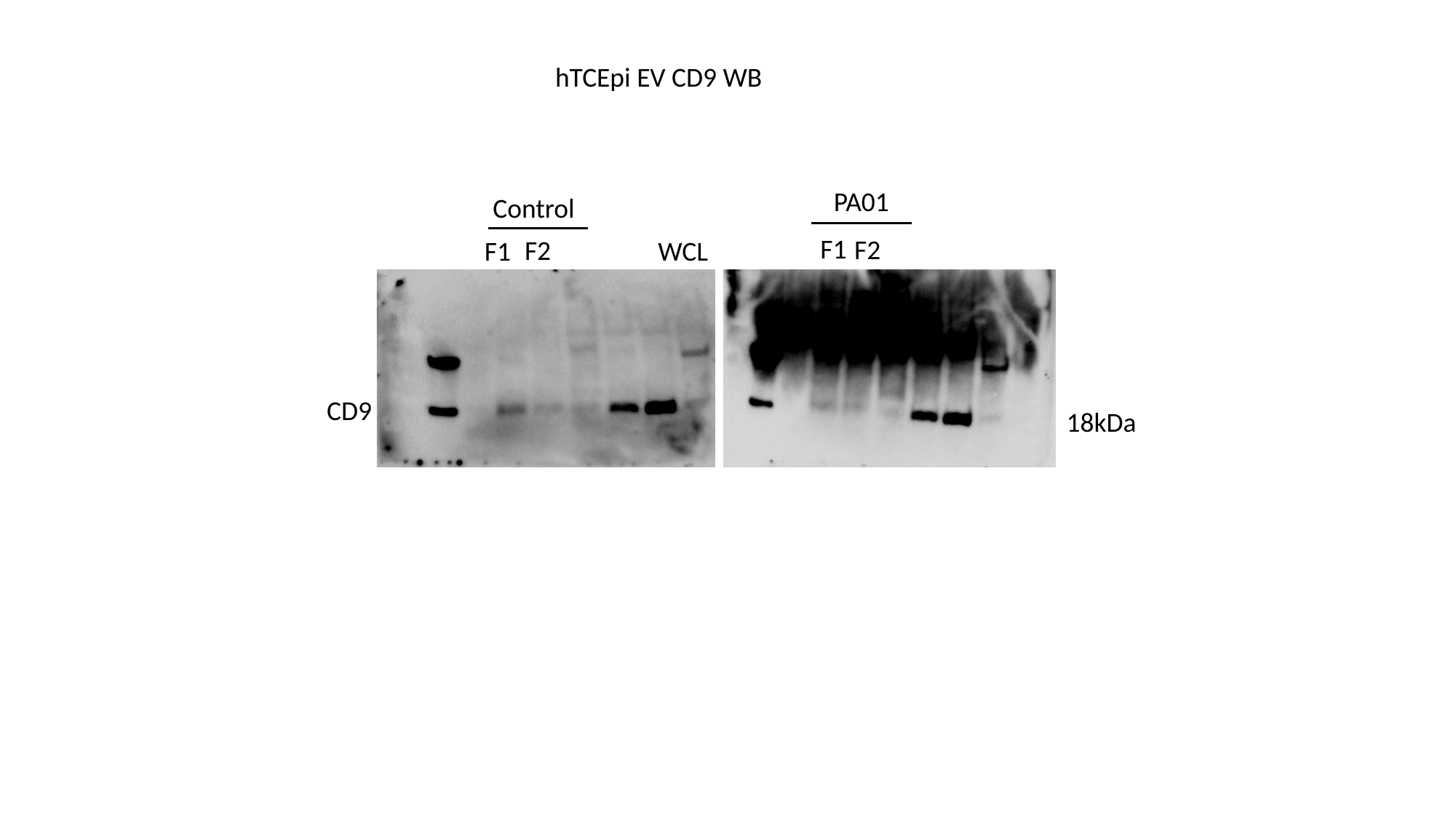

hTCEpi EV CD9 WB
PA01
Control
F1
F2
F2
F1
WCL
CD9
18kDa

## Slide 2
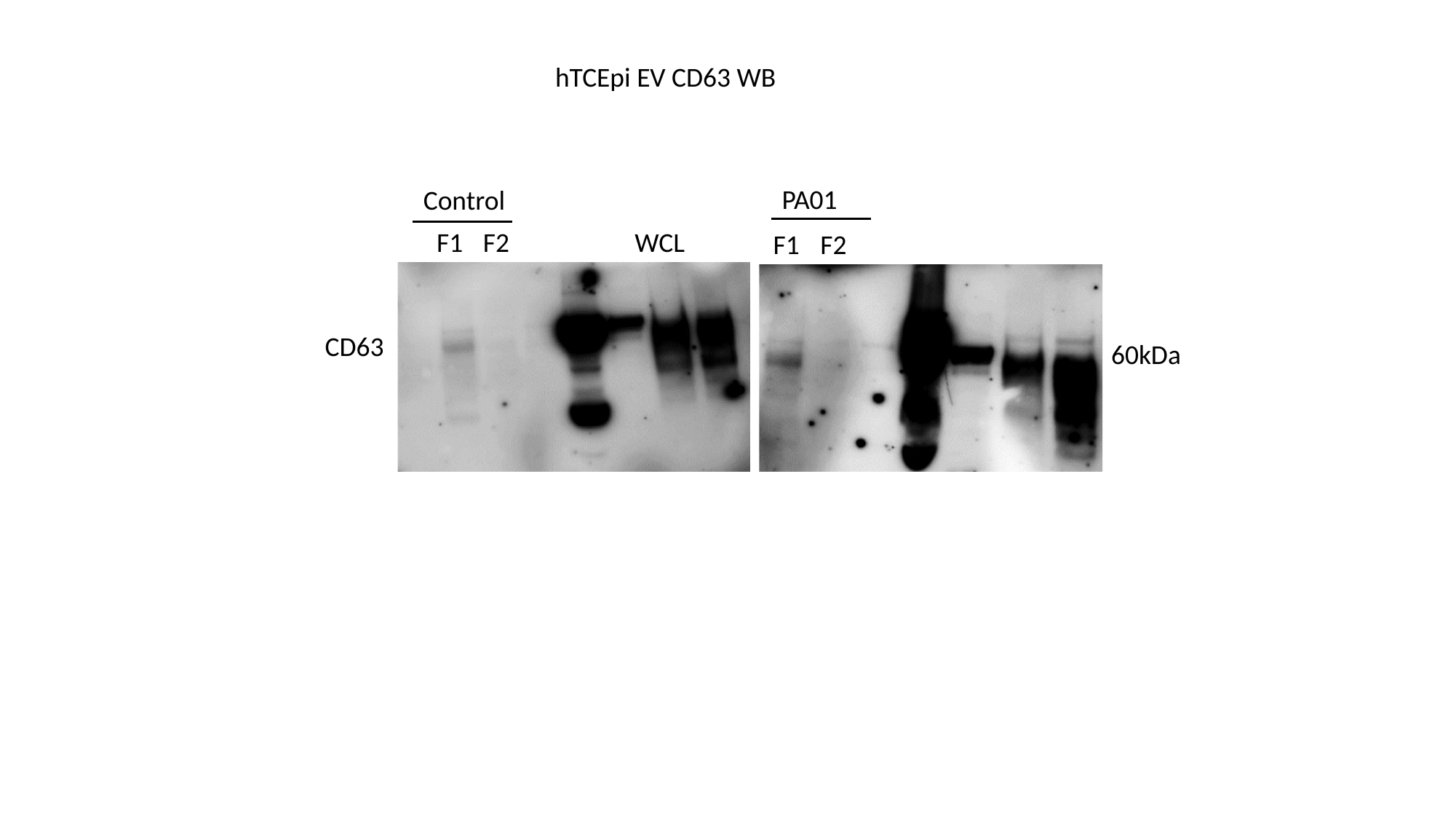

hTCEpi EV CD63 WB
PA01
Control
F2
F1
WCL
F1
F2
CD63
60kDa

## Slide 3
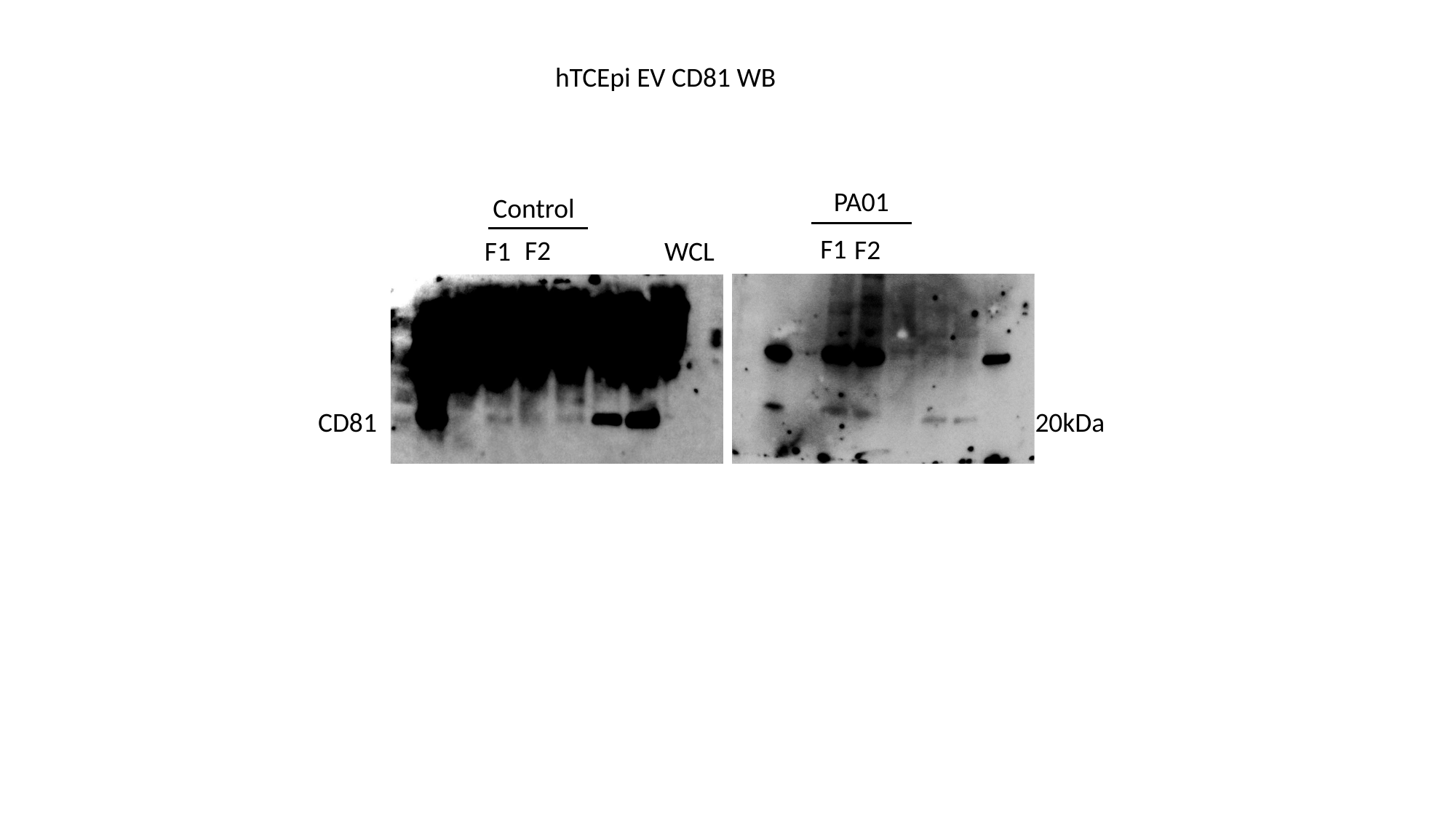

hTCEpi EV CD81 WB
PA01
Control
F1
F2
F2
F1
WCL
CD81
20kDa

## Slide 4
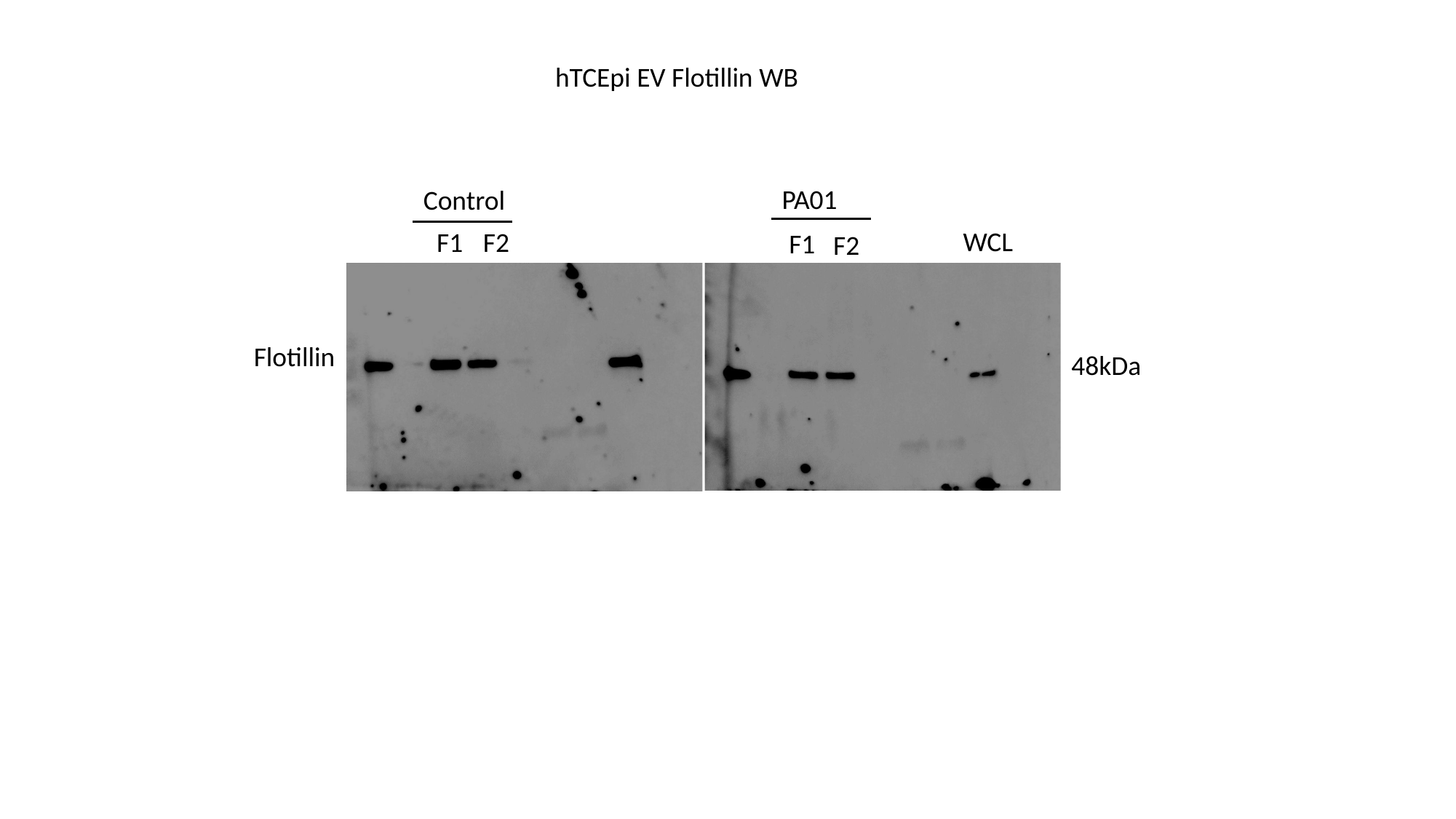

hTCEpi EV Flotillin WB
PA01
Control
WCL
F2
F1
F1
F2
Flotillin
48kDa

## Slide 5
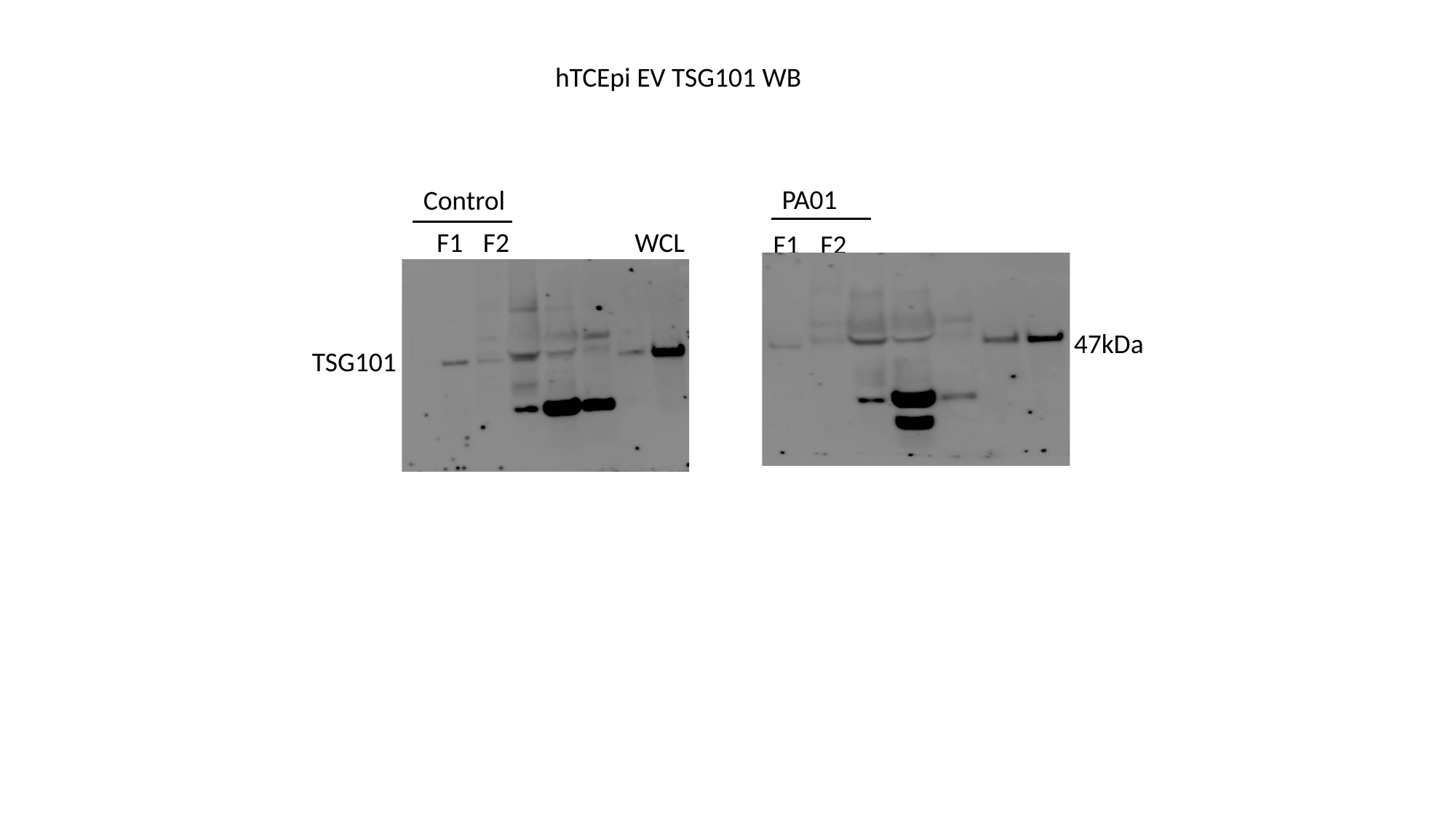

hTCEpi EV TSG101 WB
PA01
Control
F2
F1
WCL
F1
F2
47kDa
TSG101

## Slide 6
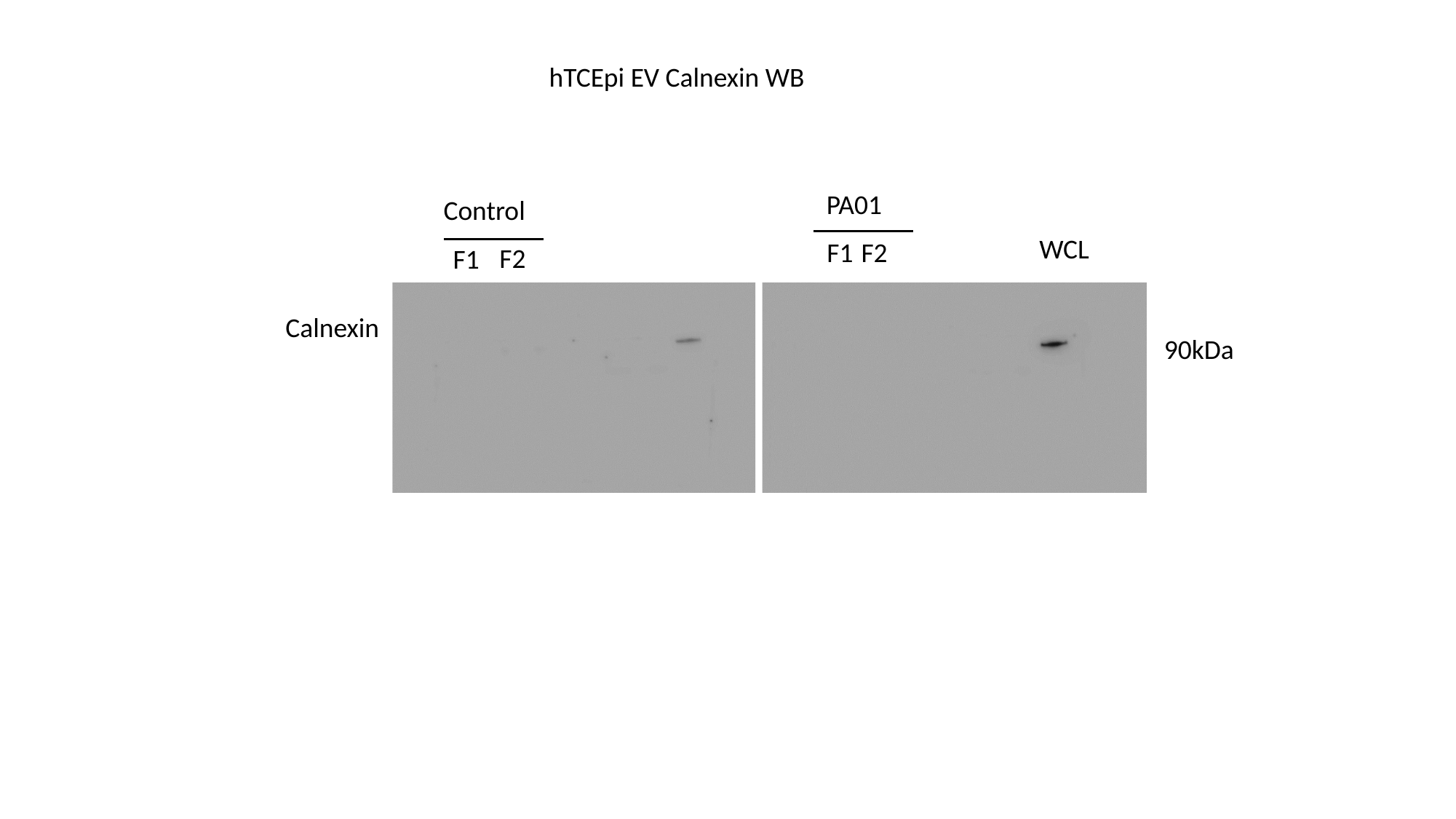

hTCEpi EV Calnexin WB
PA01
Control
WCL
F1
F2
F2
F1
Calnexin
90kDa

## Slide 7
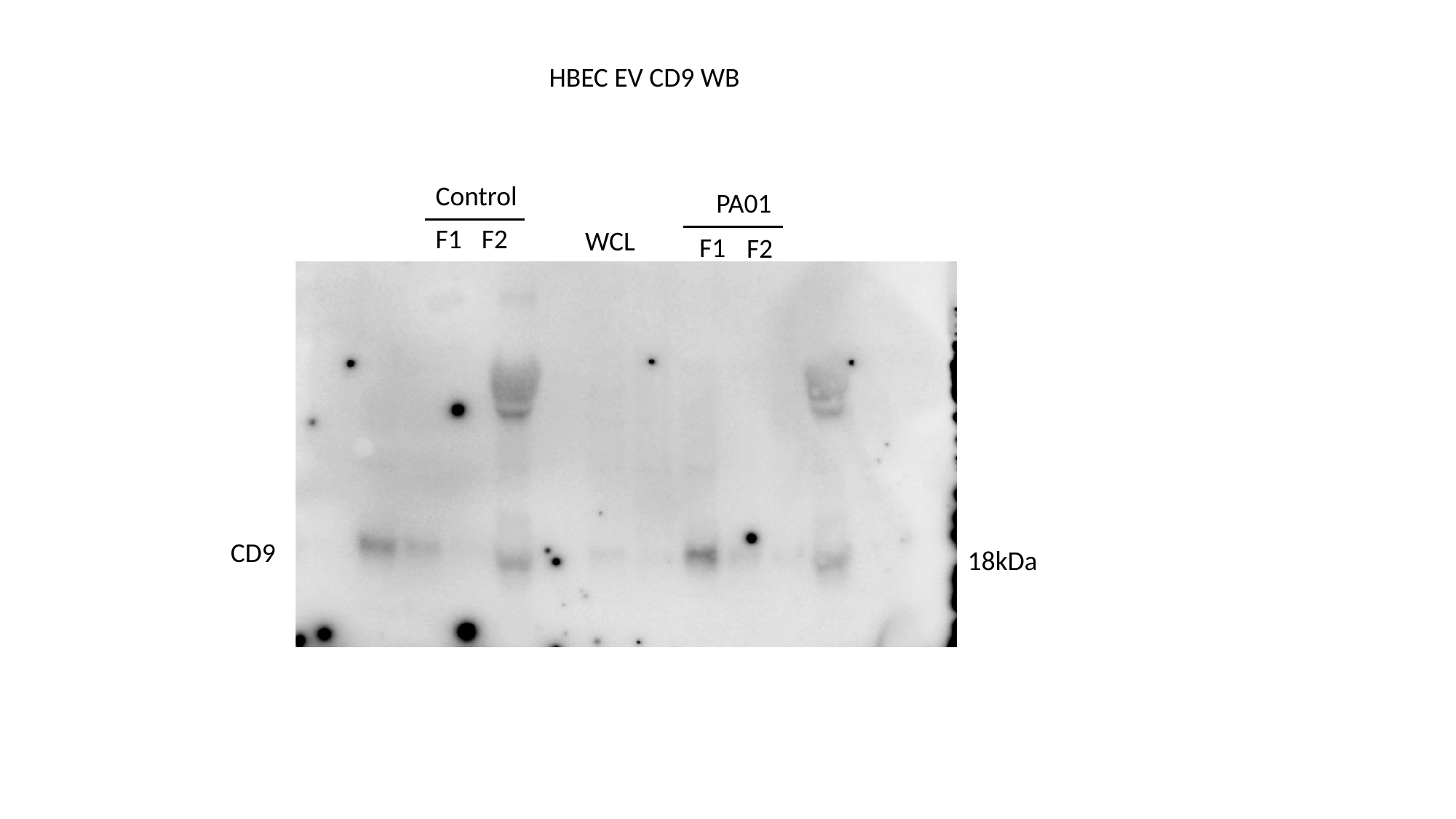

HBEC EV CD9 WB
Control
PA01
F2
F1
WCL
F1
F2
CD9
18kDa

## Slide 8
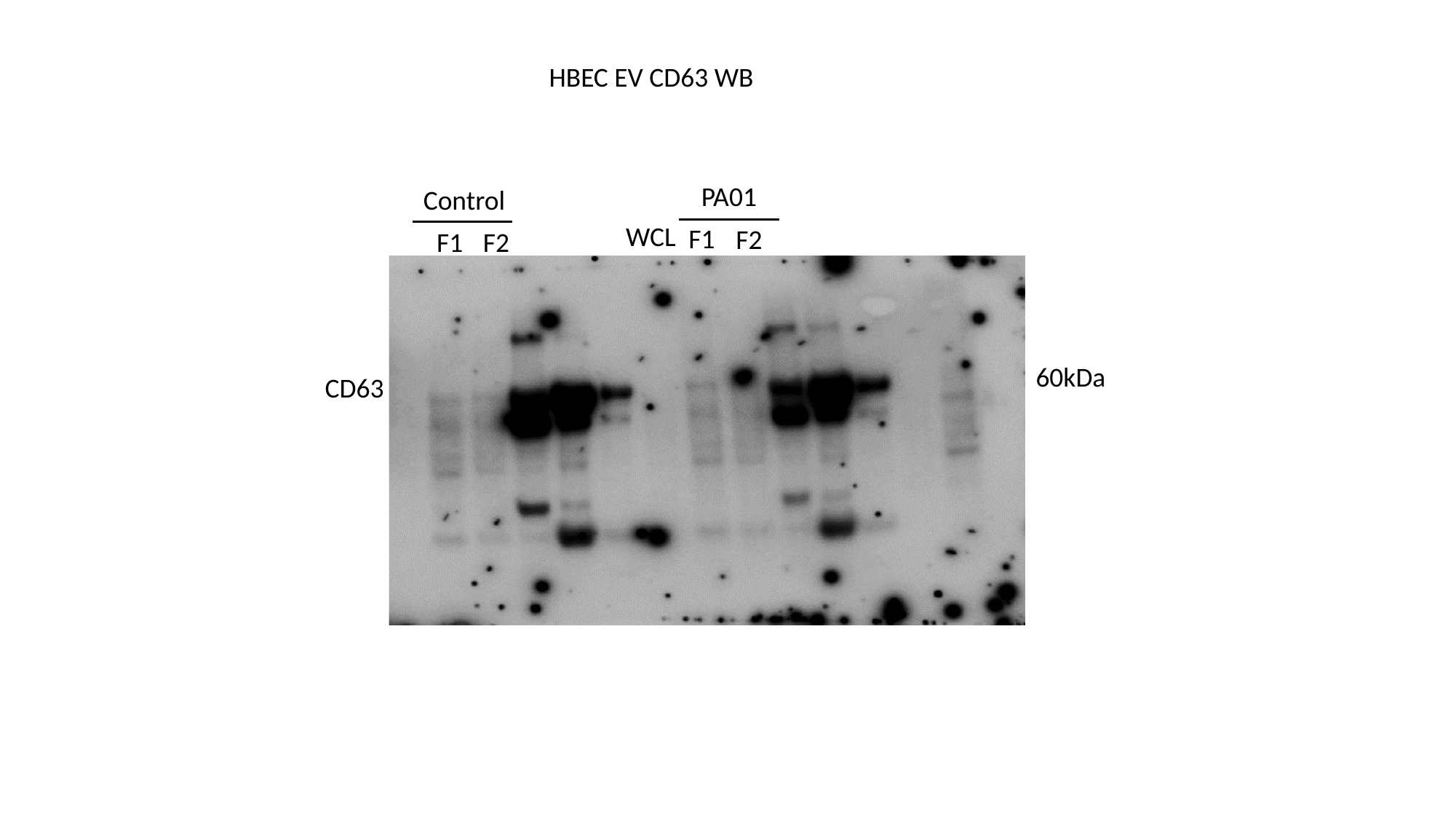

HBEC EV CD63 WB
PA01
Control
WCL
F1
F2
F2
F1
60kDa
CD63

## Slide 9
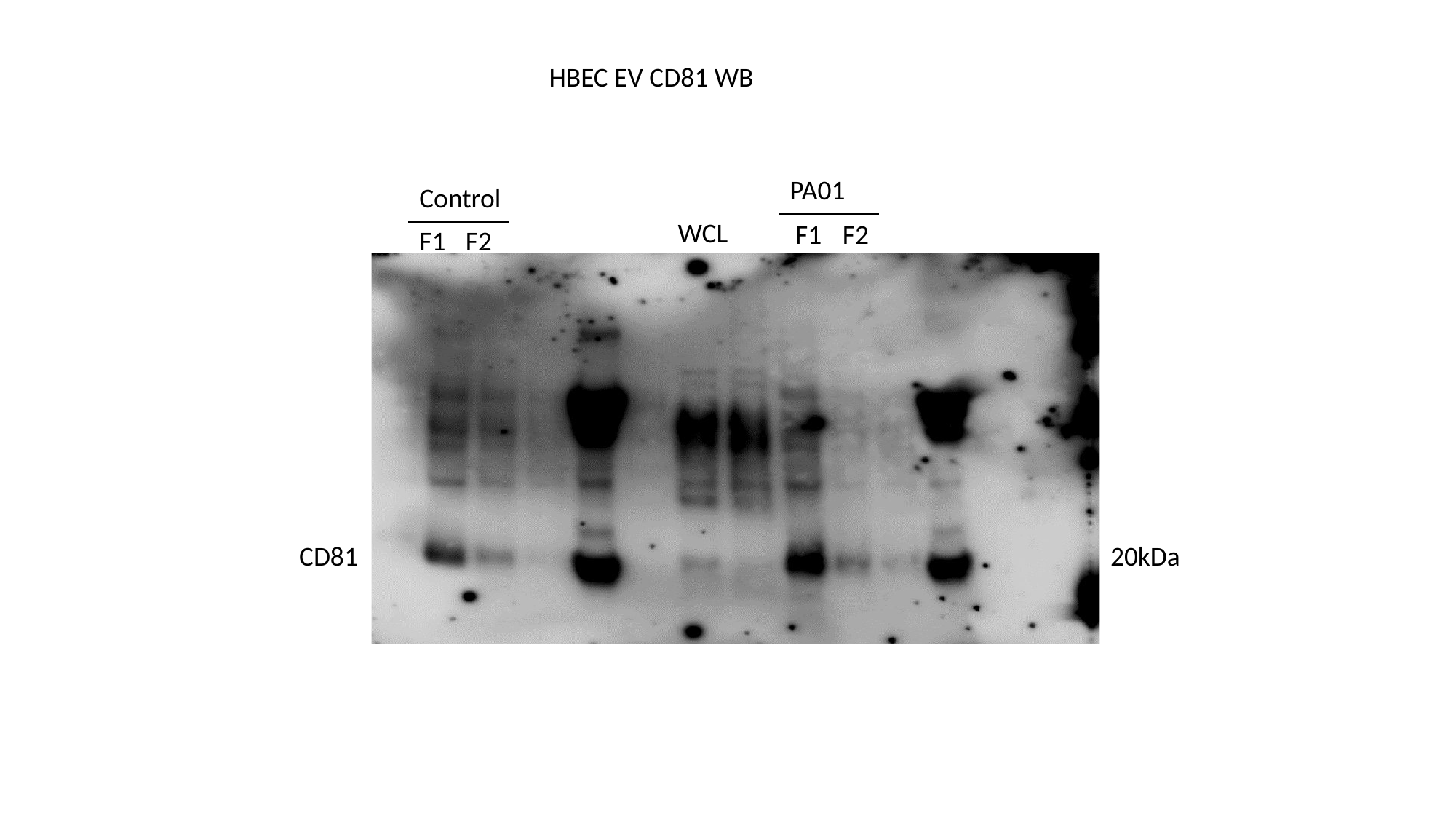

HBEC EV CD81 WB
PA01
Control
WCL
F1
F2
F2
F1
CD81
20kDa

## Slide 10
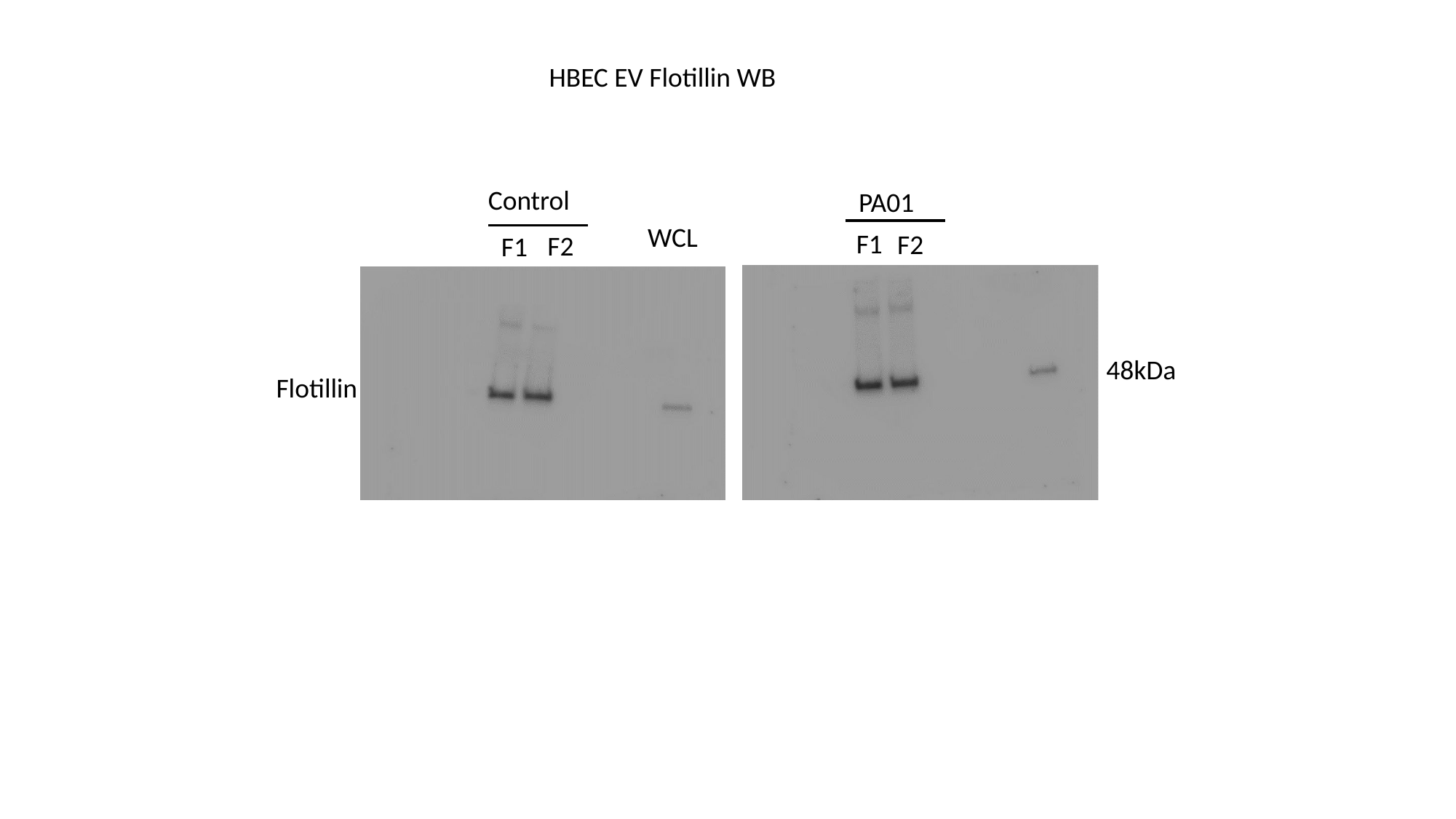

HBEC EV Flotillin WB
Control
PA01
WCL
F1
F2
F2
F1
48kDa
Flotillin

## Slide 11
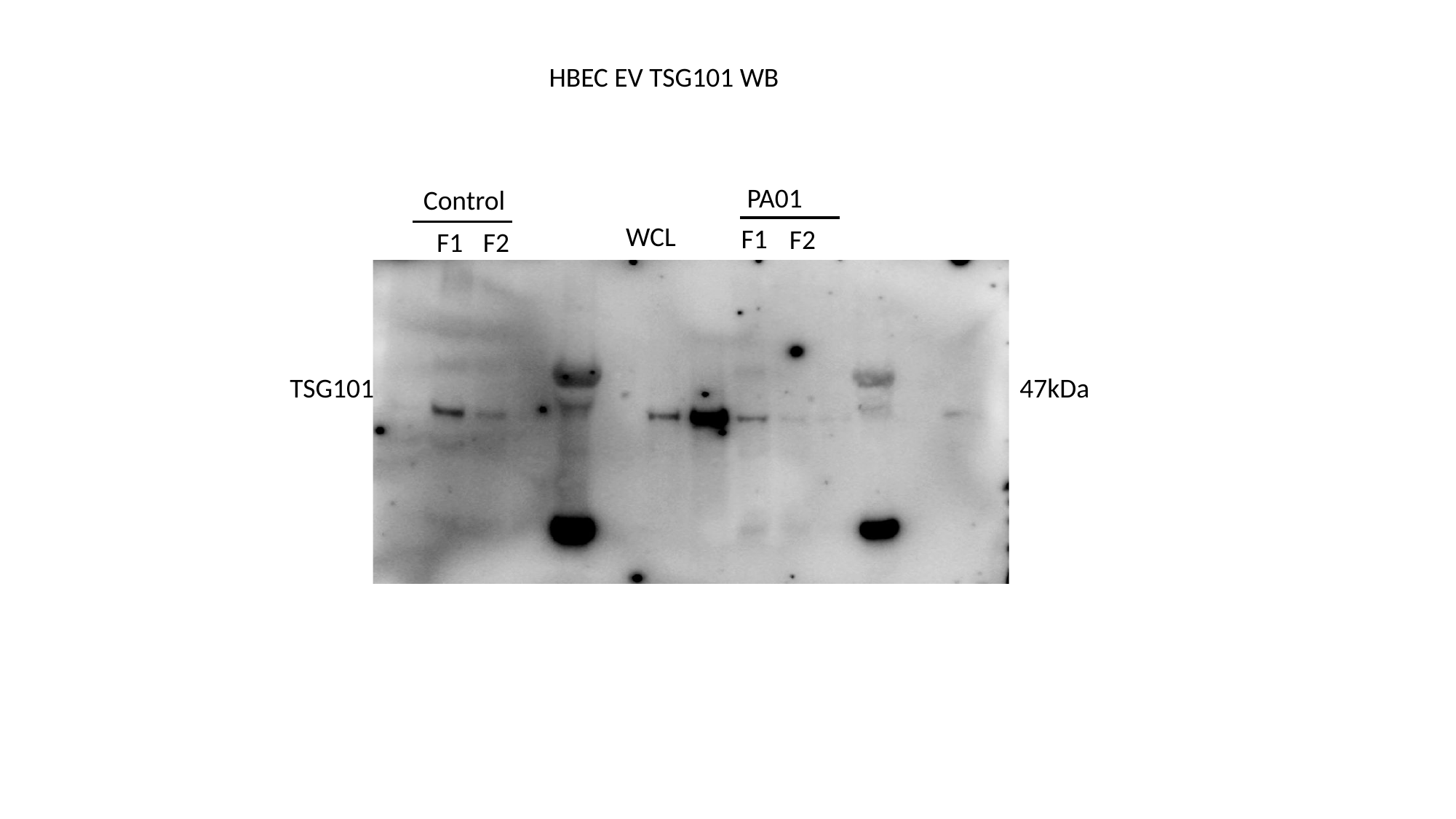

HBEC EV TSG101 WB
PA01
Control
WCL
F1
F2
F2
F1
TSG101
47kDa

## Slide 12
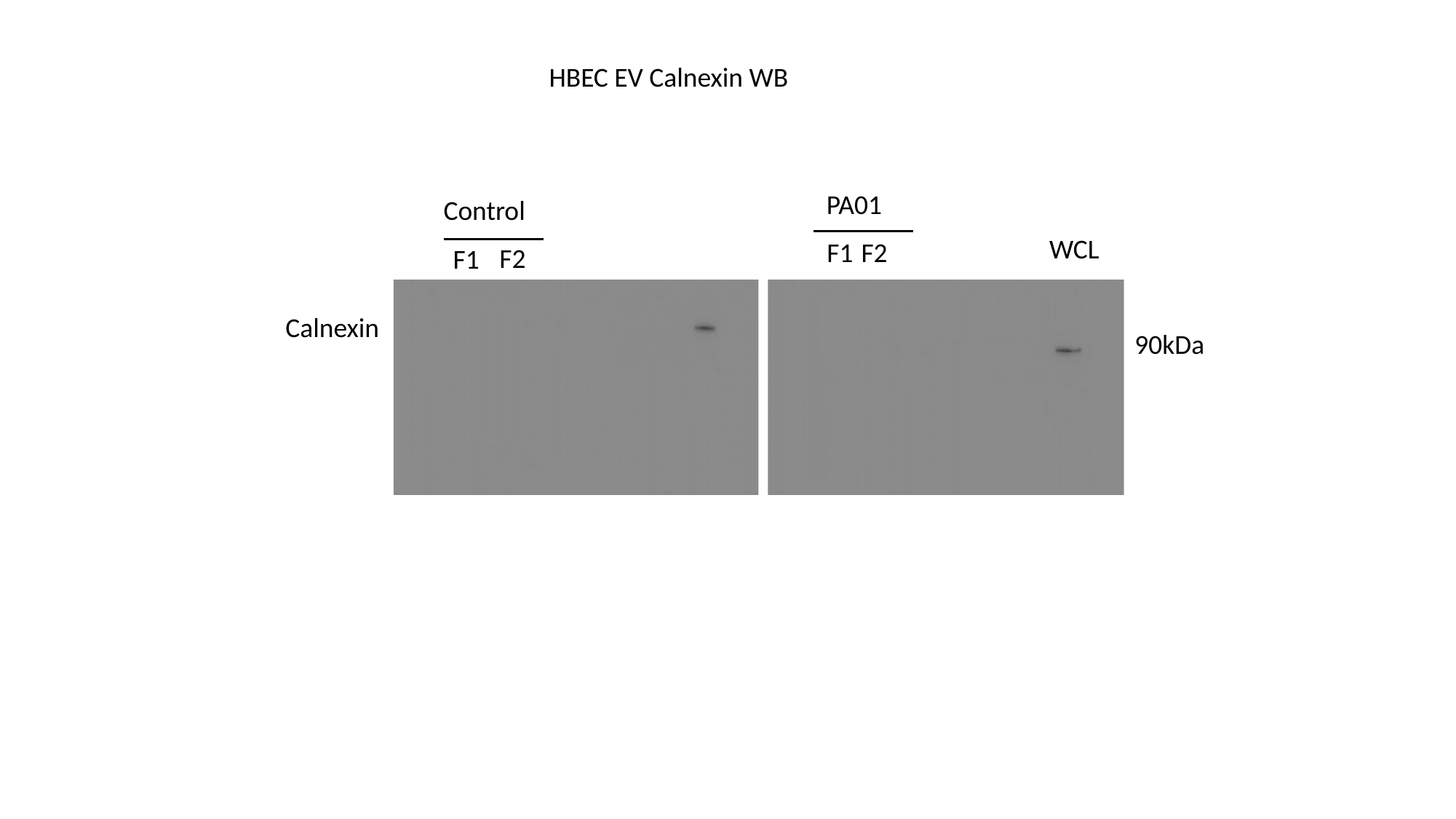

HBEC EV Calnexin WB
PA01
Control
WCL
F1
F2
F2
F1
Calnexin
90kDa
